# Catalogue of type specimens deposited in the Polychaeta Collection of the Universidad Autónoma de Nuevo Léon (Mexico)

**DOI:** 10.3897/BDJ.12.e118576

**Published:** 2024-03-12

**Authors:** María Elena García-Garza, Jesús Angel de León-González, María Ana Tovar-Hernández

**Affiliations:** 1 Universidad Autónoma de Nuevo León, Facultad de Ciencias Biológicas, Laboratorio de Sistemática, San Nicolás de los Garza, Nuevo León, Mexico Universidad Autónoma de Nuevo León, Facultad de Ciencias Biológicas, Laboratorio de Sistemática San Nicolás de los Garza, Nuevo León Mexico

**Keywords:** annelids, polychaetes, holotypes, paratypes, syntype, catalogue, UANL

## Abstract

**Background:**

In Mexico, there are six scientific collections of polychaetous annelids. The "Colección Poliquetológica" from the Universidad Autónoma de Nuevo León houses one of the three most important collections of annelids in the country, based on a number of lots and number of type materials deposited, as well as geographical coverage.

**New information:**

A catalogue of type materials of polychaete annelids housed at the “Colección Poliquetológica” from the Universidad Autónoma de Nuevo León (México) is presented for the first time. The Collection contains 37 holotypes, 174 paratypes and one syntype. These type materials are grouped in 15 families, 35 genera and 54 species of marine worms. Types were described mostly from the Mexican waters, with a low number of types from Ecuador, El Salvador, Argentina, USA, Philippines, New Caledonia and Japan.

## Introduction

The Biological Sciences Faculty from the Autonomous University of Nuevo León (UANL, Spanish acronym), houses one of the three most important collections of polychaetous annelids in Mexico. According to the most recent census that date back from 2014, the "Colección de Referencia de Bentos Costeros" from El Colegio de la Frontera Sur, Unidad Chetumal (QNR.IN.021.0497) maintained 26 holotypes, 42 paratypes and 33 syntypes. The "Colección de Anélidos Poliquetos de México" (DFE.IN.061.0598) from the Instituto de Ciencias del Mar y Limnología, UNAM housed 16 holotypes, one neotype and 28 paratypes, whereas in the "Colección Poliquetológica de la Universidad Autónoma de Nuevo León" (NL-INV-0002-05-09), 24 holotypes, 9 paratypes and one syntype were deposited (Tovar-Hernández et al. 2014).

The "Colección Poliquetológica de la Universidad Autónoma de Nuevo León" originated in 1964, when students and career professors started to collect samples along Mexican littorals during fieldwork and teaching lessons. These samples were identified and then deposited at the Laboratory of Arthropods. Unfortunately, the laboratory and part of the incipient collection was destroyed by fire in 1975.

From 1979 to 1981, Sergio Salazar-Vallejo, being a bachelor student on that time, recovered some specimens and transferred them to the Non-Arthropod Invertebrate Zoology Laboratory. He organised the collection and also provided the first catalogue being composed of 26 families, 59 genera and 74 species (Salazar-Vallejo 1981, Salazar-Vallejo 2023).

Then, from 1982 to 1985, the collection was in the charge of Jesús Angel de León González, who moved to La Paz (Baja California Sur, Mexico) for his postgraduate studies. He returned to the Autonomous University of Nuevo León in 1991 as curator, bringing with him numerous lots of polychaete worms captured by himself during oceanographic cruises, as well as sporadical samplings along the coast of the Gulf of California and the Baja California Peninsula.

**Figure 1.**
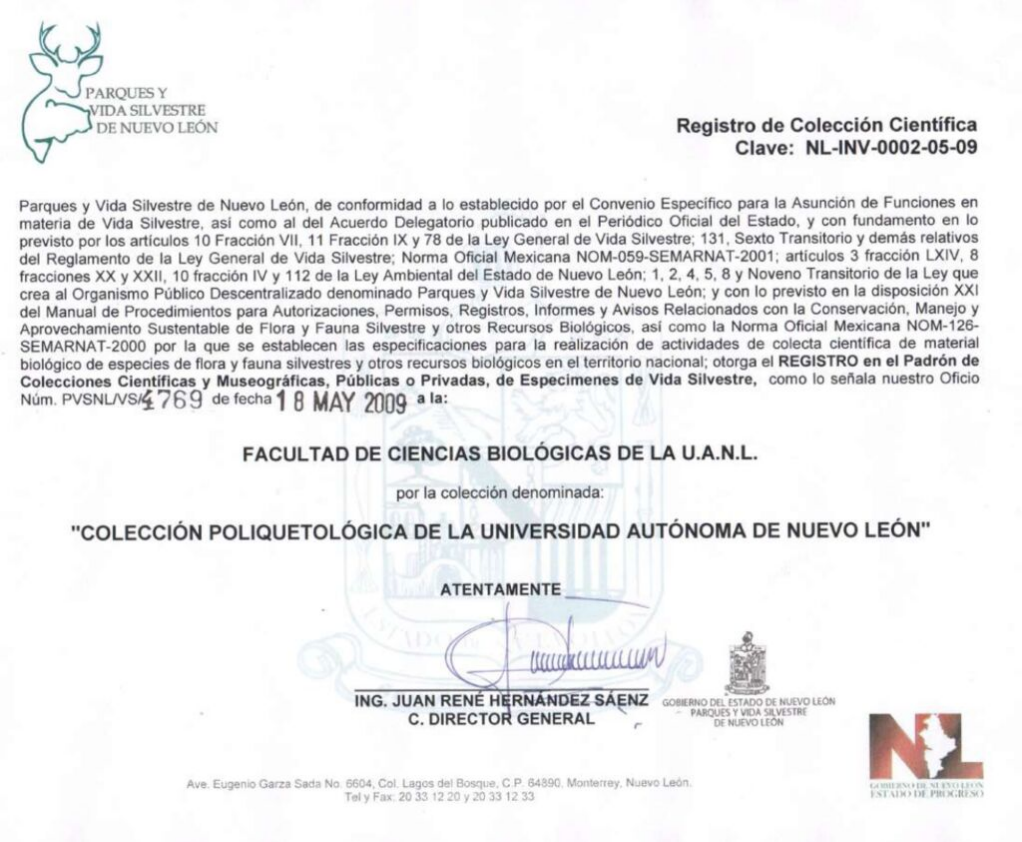
Formal record of the "Colección Poliquetológica de la Universidad Autónoma de Nuevo León".

Finally, the collection obtained formal recognition and their record in the census of Public and Private Scientific and Museographic collections for wildlife of the Secretaría del Medio Ambiente y Recursos Naturales (SEMARNAT) in May 2009. The collection officially adopted the acronym UANL (Universidad Autónoma de Nuevo León) with key number NL-INV-0002-05-09 (Fig. 1).

Currently, the collection houses 8,159 lots belonging to 51 families, 285 genera and 699 species of polychaetes. In addition, it houses type materials of annelids mostly from Mexico, but also from Ecuador, El Salvador, Argentina, USA, Philippines, New Caledonia and Japan.

In fulfilment of the International Code of Zoological Nomenclature Article 72.10, recommendation 72F (ICZN 1999), in this contribution a compendium of the type material included in the UANL collection is provided.

## Materials and methods

The catalogue of type materials is presented in alphabetical order to families, genera and species level, including accession numbers, locality, geolocation, depth, collection date and names for collectors when available in protologues. Specimens were typically fixed with formalin and stored in 70% ethanol at ambient temperature, except for *Pectinariasantii* Tovar-Hernández & de León-González, 2022 (Tovar-Hernández and de León-González 2022) that was fixed in 96% ethanol.

Geolocations of type localities are derived from the original descriptions, being considered an “original geolocation” when the authors provided the coordinates or “estimated geolocation”, where estimated using Google Earth (Google Earth 2023) from the general geographic limits described by the authors. All geolocations were converted to decimal degrees throughout PGC Coordinate Converter (The Polar Geospatial Center 2023).

References for taxonomic authorities in phylum, class, family and genera are not included; these can be consulted in WoRMS (2023). Corresponding protologues to species are provided in Suppl. material 1.

## Checklists

### Species with type materials deposited at the "Colección Poliquetológica de la Universidad Autónoma de Nuevo León"

#### Dasybranchethus pacifica

García-Garza & de León-González, 2009

07C3F45D-EBE3-5D84-8C5D-F9F2B88DFDB3

https://marinespecies.org/aphia.php/aphia.php?p=taxdetails&id=425711

##### Materials

- **Type status:** Holotype. **Occurrence:** catalogNumber: UANL 6336; recordedBy: María E. García-Garza | Jesús A. de León-González; occurrenceID: 3BBF8D08-8030-5CFC-A7B6-305E4AD7CC38; **Taxon:** kingdom: Animalia; phylum: Annelida; class: Polychaeta; order: Scolecida; family: Capitellidae; genus: Dasybranchetus; **Location:** continent: North America; waterBody: Gulf of California; country: Mexico; countryCode: MX; stateProvince: Baja California Sur; municipality: Concepción Bay; locality: Santispac; maximumDepthInMeters: 1; decimalLatitude: 26.762; decimalLongitude: -111.891944; **Event:** eventDate: 26VI2005; **Record Level:** institutionCode: UANL; collectionCode: NL-INV-0002-05-09
- **Type status:** Paratype. **Occurrence:** catalogNumber: UANL 6337; recordedBy: María E. García-Garza | Jesús A. de León-González; individualCount: 1; occurrenceID: 8A07C42C-5257-5AB1-A536-839C2B633A39; **Taxon:** kingdom: Animalia; phylum: Annelida; class: Polychaeta; order: Scolecida; family: Capitellidae; genus: Dasybranchetus; **Location:** continent: North America; waterBody: Gulf of California; country: Mexico; countryCode: MX; stateProvince: Baja California Sur; municipality: Concepción Bay; locality: El Quemadito; maximumDepthInMeters: 1; decimalLatitude: 26.759194; decimalLongitude: -111.876806; **Event:** eventDate: 26VI2005; **Record Level:** institutionCode: UANL; collectionCode: NL-INV-0002-05-09

#### Notodasus harrisae

García-Garza, Hernández-Valdez & de León-González, 2009

FA3E0956-A169-51AA-8748-C540D2F1A47C

http://www.marinespecies.org/aphia.php?p=taxdetails&id=590097

##### Materials

- **Type status:** Holotype. **Occurrence:** catalogNumber: UANL 6510; recordedBy: María E. García-Garza | Jesús A. de León-González; occurrenceID: EBE6E3C1-908F-5AA7-A484-3EC495BB589E; **Taxon:** kingdom: Animalia; phylum: Annelida; class: Polychaeta; order: Scolecida; family: Capitellidae; genus: Notodasus; **Location:** continent: North America; waterBody: Gulf of California; country: Mexico; countryCode: MX; stateProvince: Baja California Sur; municipality: La Paz Bay; locality: El Tesoro; maximumDepthInMeters: 1; decimalLatitude: 24.254472; decimalLongitude: -110.315389; **Event:** eventDate: 1VIII2006; **Record Level:** institutionCode: UANL; collectionCode: NL-INV-0002-05-09

#### Notodasus hartmanae

García-Garza, Hernández-Valdez & de León-González, 2009

90D5168B-3426-54A0-B41B-7F867B5F5FEA

https://www.marinespecies.org/aphia.php?p=taxdetails&id=590098

##### Materials

- **Type status:** Holotype. **Occurrence:** catalogNumber: UANL 6513; recordedBy: Jesús A. de León-González; occurrenceID: 6C9646ED-4F4C-5FB1-BBB4-190CAAC1E516; **Taxon:** kingdom: Animalia; phylum: Annelida; class: Polychaeta; order: Scolecida; family: Capitellidae; genus: Notodasus; **Location:** continent: North America; waterBody: Tropical Eastern Pacific; country: Mexico; countryCode: MX; stateProvince: Chiapas; municipality: Paredón; locality: Mar Muerto; maximumDepthInMeters: 0.5; decimalLatitude: 16.057222; decimalLongitude: -93.87611099999999; **Event:** eventDate: 14IV2008; **Record Level:** institutionCode: UANL; collectionCode: NL-INV-0002-05-09
- **Type status:** Paratype. **Occurrence:** catalogNumber: UANL 6514; recordedBy: Jesús A. de León-González; individualCount: 6; occurrenceID: 6A3E248D-58D4-5C24-BE0B-5E50D3CC1F89; **Taxon:** kingdom: Animalia; phylum: Annelida; class: Polychaeta; order: Scolecida; family: Capitellidae; genus: Notodasus; **Location:** continent: North America; waterBody: Tropical Eastern Pacific; country: Mexico; countryCode: MX; stateProvince: Chiapas; municipality: Paredón; locality: Mar Muerto; maximumDepthInMeters: 0.5; decimalLatitude: 16.057222; decimalLongitude: -93.87611099999999; **Event:** eventDate: 14IV2008; **Record Level:** institutionCode: UANL; collectionCode: NL-INV-0002-05-09

#### Notodasus kristiani

García-Garza, Hernández-Valdez & de León-González, 2009

08995D2B-7B4D-5397-B1BF-48426E194050

https://www.marinespecies.org/aphia.php?p=taxdetails&id=590099

##### Materials

- **Type status:** Holotype. **Occurrence:** catalogNumber: UANL 6515; recordedBy: María E. García-Garza | Jesús A. de León-González; occurrenceID: FE82D625-C785-577E-B951-49F7C28A1910; **Taxon:** kingdom: Animalia; phylum: Annelida; class: Polychaeta; order: Scolecida; family: Capitellidae; genus: Notodasus; **Location:** continent: North America; waterBody: Gulf of California; country: Mexico; countryCode: MX; stateProvince: Sonora; municipality: Guaymas; locality: Varadero; maximumDepthInMeters: 1; decimalLatitude: 27.901194; decimalLongitude: -110.868806; **Event:** eventDate: 01VII2005; **Record Level:** institutionCode: UANL; collectionCode: NL-INV-0002-05-09
- **Type status:** Paratype. **Occurrence:** catalogNumber: UANL 6517; recordedBy: María E. García-Garza | Jesús A. de León-González; individualCount: 20; occurrenceID: EFD17A4B-5B2C-531C-AB9E-DF9B5DDFCBB1; **Taxon:** kingdom: Animalia; phylum: Annelida; class: Polychaeta; order: Scolecida; family: Capitellidae; genus: Notodasus; **Location:** continent: North America; waterBody: Gulf of California; country: Mexico; countryCode: MX; stateProvince: Sonora; municipality: Guaymas; locality: Varadero; maximumDepthInMeters: 1; decimalLatitude: 27.901194; decimalLongitude: -110.868806; **Event:** eventDate: 01VII2005; **Record Level:** institutionCode: UANL; collectionCode: NL-INV-0002-05-09

#### Notodasus salazari

García-Garza, Hernández-Valdez & de León-González, 2009

8D19D339-994E-5CBE-B3F7-EDA4ED64F9F5

https://www.marinespecies.org/aphia.php?p=taxdetails&id=590100

##### Materials

- **Type status:** Holotype. **Occurrence:** catalogNumber: UANL 6518; recordedBy: María E. García-Garza | Sergio I. Salazar-Vallejo; occurrenceID: 7D330129-ED9B-5068-B626-F5A5E382AF2A; **Taxon:** kingdom: Animalia; phylum: Annelida; class: Polychaeta; order: Scolecida; family: Capitellidae; genus: Notodasus; **Location:** continent: North America; waterBody: Caribbean Sea; country: Mexico; countryCode: MX; stateProvince: Quintana Roo; municipality: Isla Contoy; locality: Isla Contoy; maximumDepthInMeters: 0.9; decimalLatitude: 21.460517; decimalLongitude: -86.78581699999999; **Event:** eventDate: 21II2008; **Record Level:** institutionCode: UANL; collectionCode: NL-INV-0002-05-09

#### Notomastus fauchaldi

García-Garza & de León-González, 2015

EC975BA6-1354-522D-9489-F799CE8062A4

https://www.marinespecies.org/aphia.php?p=taxdetails&id=852603

##### Materials

- **Type status:** Holotype. **Occurrence:** catalogNumber: UANL 6537; recordedBy: V.D. Hernández Valdez; occurrenceID: 3A3E221D-A0D3-5C3C-AB27-59ECF2DC027C; **Taxon:** kingdom: Animalia; phylum: Annelida; class: Polychaeta; order: Scolecida; family: Capitellidae; genus: Notomastus; **Location:** continent: North America; waterBody: Gulf of California; country: Mexico; countryCode: MX; stateProvince: Baja California Sur; municipality: Ensenada de La Paz; locality: Bahía de la Paz; maximumDepthInMeters: 2; decimalLatitude: 24.127028; decimalLongitude: -110.419611; **Event:** eventDate: 01VI2006; **Record Level:** institutionCode: UANL; collectionCode: NL-INV-0002-05-09
- **Type status:** Paratype. **Occurrence:** catalogNumber: UANL 6538; recordedBy: V.D. Hernández Valdez; individualCount: 1; occurrenceID: B1C20A37-DC1A-557B-BEA7-F887384E9B40; **Taxon:** kingdom: Animalia; phylum: Annelida; class: Polychaeta; order: Scolecida; family: Capitellidae; genus: Notomastus; **Location:** continent: North America; waterBody: Gulf of California; country: Mexico; countryCode: MX; stateProvince: Baja California Sur; municipality: Ensenada de La Paz; locality: Bahía de la Paz; maximumDepthInMeters: 2; decimalLatitude: 24.127028; decimalLongitude: -110.419611; **Event:** eventDate: 01VI2006; **Record Level:** institutionCode: UANL; collectionCode: NL-INV-0002-05-09
- **Type status:** Paratype. **Occurrence:** catalogNumber: UANL 6539; recordedBy: V.D. Hernández Valdez; individualCount: 1; occurrenceID: 8D3DCB00-D64E-5428-B82F-EAF4D14975C5; **Taxon:** kingdom: Animalia; phylum: Annelida; class: Polychaeta; order: Scolecida; family: Capitellidae; genus: Notomastus; **Location:** continent: North America; waterBody: Gulf of California; country: Mexico; countryCode: MX; stateProvince: Baja California Sur; municipality: Ensenada de La Paz; locality: Bahía de la Paz; maximumDepthInMeters: 2; decimalLatitude: 24.127028; decimalLongitude: -110.419611; **Event:** eventDate: 01VI2006; **Record Level:** institutionCode: UANL; collectionCode: NL-INV-0002-05-09

#### Notomastus landini

García-Garza & de León-González, 2015

0F19D6F0-EB7C-525A-9AAD-C6B93C96D7F4

https://www.marinespecies.org/aphia.php?p=taxdetails&id=852601

##### Materials

- **Type status:** Holotype. **Occurrence:** catalogNumber: UANL 6546; recordedBy: María E. García-Garza | Jesús A. de León-González; occurrenceID: C72D756B-5C93-5FFB-B353-0C72BD73D6EF; **Taxon:** kingdom: Animalia; phylum: Annelida; class: Polychaeta; order: Scolecida; family: Capitellidae; genus: Notomastus; **Location:** continent: North America; waterBody: Gulf of California; country: Mexico; countryCode: MX; stateProvince: Baja California Sur; municipality: Concepción Bay; locality: Los Cocos; maximumDepthInMeters: 1; decimalLatitude: 26.744194; decimalLongitude: -111.898722; **Event:** eventDate: 25VI2006; **Record Level:** institutionCode: UANL; collectionCode: NL-INV-0002-05-09
- **Type status:** Paratype. **Occurrence:** catalogNumber: UANL 7846; recordedBy: María E. García-Garza | Jesús A. de León-González; individualCount: 4; occurrenceID: 3F61113F-F686-5870-9EA5-14F938649E7F; **Taxon:** kingdom: Animalia; phylum: Annelida; class: Polychaeta; order: Scolecida; family: Capitellidae; genus: Notomastus; **Location:** continent: North America; waterBody: Gulf of California; country: Mexico; countryCode: MX; stateProvince: Baja California Sur; municipality: Concepción Bay; locality: Los Cocos; maximumDepthInMeters: 1; decimalLatitude: 26.744194; decimalLongitude: -111.898722; **Event:** eventDate: 25VI2006; **Record Level:** institutionCode: UANL; collectionCode: NL-INV-0002-05-09

#### Notomastus lobulatus

García-Garza & de León-González, 2015

A83D6E9D-F418-5D20-972D-6FAC5DBC31FB

https://www.marinespecies.org/aphia.php?p=taxdetails&id=852602

##### Materials

- **Type status:** Holotype. **Occurrence:** catalogNumber: UANL 7847; recordedBy: Nuria Méndez-Ubach; occurrenceID: 358C24E9-30EB-5343-9250-F0669326A3DC; **Taxon:** kingdom: Animalia; phylum: Annelida; class: Polychaeta; order: Scolecida; family: Capitellidae; genus: Notomastus; **Location:** continent: North America; waterBody: Gulf of California; country: Mexico; countryCode: MX; stateProvince: Sinaloa; municipality: Off Sinaloa coast; locality: Open sea; minimumDepthInMeters: 1200; maximumDepthInMeters: 1274; decimalLatitude: 24.938333; decimalLongitude: -109.196667; **Event:** eventDate: 26VIII2000; **Record Level:** institutionCode: UANL; collectionCode: NL-INV-0002-05-09

#### Notomastus mazatlanensis

García-Garza, de León-González & Tovar-Hernández, 2019

21CFFD47-83ED-50EA-90D5-2C551DFF0FD5

https://www.marinespecies.org/aphia.php?p=taxdetails&id=1338748

##### Materials

- **Type status:** Holotype. **Occurrence:** catalogNumber: UANL 8128; recordedBy: María E. García-Garza | María A. Tovar-Hernández | Jesús A. de León-González; occurrenceID: 984540C7-5395-522E-9B9B-015CB593D872; **Taxon:** kingdom: Animalia; phylum: Annelida; class: Polychaeta; order: Scolecida; family: Capitellidae; genus: Notomastus; **Location:** continent: North America; waterBody: Gulf of California; country: Mexico; countryCode: MX; stateProvince: Sinaloa; municipality: Mazatlán; locality: Marina Mazatlán; maximumDepthInMeters: 6; decimalLatitude: 23.26725; decimalLongitude: -106.454028; **Event:** eventDate: 05IX2018; **Record Level:** institutionCode: UANL; collectionCode: NL-INV-0002-05-09
- **Type status:** Paratype. **Occurrence:** catalogNumber: UANL 8129; recordedBy: María E. García-Garza | María A. Tovar-Hernández | Jesús A. de León-González; individualCount: 4; occurrenceID: 7A75CD4B-D44D-58AA-93B2-ACB29FEF34CE; **Taxon:** kingdom: Animalia; phylum: Annelida; class: Polychaeta; order: Scolecida; family: Capitellidae; genus: Notomastus; **Location:** continent: North America; waterBody: Gulf of California; country: Mexico; countryCode: MX; stateProvince: Sinaloa; municipality: Mazatlán; locality: Marina Mazatlán; maximumDepthInMeters: 6; decimalLatitude: 13.168667; decimalLongitude: -87.72283299999999; **Event:** eventDate: 11X2015; **Record Level:** institutionCode: UANL; collectionCode: NL-INV-0002-05-09

#### Eunice chicasi

de León-González, Rivera & Romero, 2004

9F1C0066-55D6-505D-B2DB-481143051D7F

https://www.marinespecies.org/aphia.php?p=taxdetails&id=327656

##### Materials

- **Type status:** Holotype. **Occurrence:** catalogNumber: UANL 5488; recordedBy: Carlos G. Rivera | Mayra Y. Romero; occurrenceID: 6D9E9329-35CB-51CE-BDCE-BB64C462B323; **Taxon:** kingdom: Animalia; phylum: Annelida; class: Polychaeta; order: Eunicida; family: Eunicidae; genus: Eunice; **Location:** continent: North America; waterBody: Tropical Eastern Pacific; country: El Salvador; countryCode: SV; locality: Meanguera Island; maximumDepthInMeters: 4; decimalLatitude: 19.1885; decimalLongitude: -96.118528; **Event:** eventDate: 07III2001; **Record Level:** institutionCode: UANL; collectionCode: NL-INV-0002-05-09

#### Eunice romanvivesi

de León-González & Díaz Castañeda, 2006

23238510-A5DD-5995-B5AE-103F5773AF03

https://www.marinespecies.org/aphia.php?p=taxdetails&id=991791

##### Materials

- **Type status:** Holotype. **Occurrence:** catalogNumber: UANL 6327; recordedBy: Victoria Díaz Castañeda; occurrenceID: 1FC41570-1557-550A-9CEC-A3C6783988EF; **Taxon:** kingdom: Animalia; phylum: Annelida; class: Polychaeta; order: Eunicida; family: Eunicidae; genus: Eunice; **Location:** continent: North America; waterBody: Gulf of Mexico; country: Mexico; countryCode: MX; stateProvince: Veracruz; locality: Hornos Reef; maximumDepthInMeters: 3; decimalLatitude: 19.1885; decimalLongitude: -96.118528; **Event:** eventDate: 14VII2002; **Record Level:** institutionCode: UANL; collectionCode: NL-INV-0002-05-09
- **Type status:** Paratype. **Occurrence:** catalogNumber: UANL 6328; recordedBy: Victoria Díaz Castañeda; individualCount: 2; occurrenceID: DE3ADAF9-154B-5AEA-A1FB-085CC023581F; **Taxon:** kingdom: Animalia; phylum: Annelida; class: Polychaeta; order: Eunicida; family: Eunicidae; genus: Eunice; **Location:** continent: North America; waterBody: Gulf of Mexico; country: Mexico; countryCode: MX; stateProvince: Veracruz; locality: Hornos Reef; maximumDepthInMeters: 3; decimalLatitude: 13.1675; decimalLongitude: -87.724; **Event:** eventDate: 30V2003; **Record Level:** institutionCode: UANL; collectionCode: NL-INV-0002-05-09

#### Eunice salvadorensis

de León-González, Rivera & Romero, 2004

1B19C0E2-4F57-5A8B-B428-9E79F6DBBDCC

https://www.marinespecies.org/aphia.php?p=taxdetails&id=327789

##### Materials

- **Type status:** Holotype. **Occurrence:** catalogNumber: UANL 5489; recordedBy: Carlos G. Rivera | Mayra Y. Romero; occurrenceID: C6CC74E8-86A0-5370-BD6E-F642E8914B2B; **Taxon:** kingdom: Animalia; phylum: Annelida; class: Polychaeta; order: Eunicida; family: Eunicidae; genus: Eunice; **Location:** continent: North America; waterBody: Tropical Eastern Pacific; country: El Salvador; countryCode: SV; stateProvince: Fonseca Gulf; locality: Fonseca Gulf; maximumDepthInMeters: 5; decimalLatitude: 19.181639; decimalLongitude: -96.12261100000001; **Event:** eventDate: 11III2001; **Record Level:** institutionCode: UANL; collectionCode: NL-INV-0002-05-09

#### Treadwellphysa veracruzensis

(de León-González & Díaz Castañeda, 2006)

E202FB64-D7EC-5952-A857-0D073027730B

https://www.marinespecies.org/aphia.php?p=taxdetails&id=991754

##### Materials

- **Type status:** Holotype. **Occurrence:** catalogNumber: UANL 6329; recordedBy: Victoria Díaz Castañeda; occurrenceID: 8383450D-E7B6-5788-BEC9-1A1821A3830D; **Taxon:** kingdom: Animalia; phylum: Annelida; class: Polychaeta; order: Eunicida; family: Eunicidae; genus: Treadwellphysa; **Location:** continent: North America; waterBody: Gulf of Mexico; country: Mexico; countryCode: MX; stateProvince: Veracruz; locality: Villa del Mar; decimalLatitude: 19.181639; decimalLongitude: -96.12261100000001; **Event:** eventDate: 11VIII1999; **Record Level:** institutionCode: UANL; collectionCode: NL-INV-0002-05-09
- **Type status:** Paratype. **Occurrence:** catalogNumber: UANL 6330; recordedBy: Victoria Díaz Castañeda; individualCount: 7; occurrenceID: 8C714981-BDA5-5BC0-B569-0F493F2A0E0B; **Taxon:** kingdom: Animalia; phylum: Annelida; class: Polychaeta; order: Eunicida; family: Eunicidae; genus: Treadwellphysa; **Location:** continent: North America; waterBody: Gulf of Mexico; country: Mexico; countryCode: MX; stateProvince: Veracruz; locality: Villa del Mar; decimalLatitude: 19.181639; decimalLongitude: -96.12261100000001; **Event:** eventDate: 11VIII1999; **Record Level:** institutionCode: UANL; collectionCode: NL-INV-0002-05-09

#### Trophoniella reishi

Salazar-Vallejo, 2012

B23D4ABE-AD2F-510D-9F2E-DE5677844354

https://www.marinespecies.org/aphia.php?p=taxdetails&id=1583930

##### Materials

- **Type status:** Paratype. **Occurrence:** catalogNumber: UANL 8155; recordedBy: Jesús A. de León-González; individualCount: 1; occurrenceID: EB30ECE1-89BD-5E6E-97DB-1AB428AC0DE8; **Taxon:** kingdom: Animalia; phylum: Annelida; class: Polychaeta; order: Terebellidae; family: Flabelligeridae; genus: Trophoniella; **Location:** continent: North America; waterBody: Gulf of California; country: Mexico; countryCode: MX; stateProvince: Baja California Sur; locality: Isla Coronado; maximumDepthInMeters: 1; decimalLatitude: 26.111753; decimalLongitude: -111.284406; **Event:** eventDate: 15X1998; habitat: Intertidal; **Record Level:** institutionCode: UANL; collectionCode: NL-INV-0002-05-09

#### Trophoniella salazarae

Salazar-Vallejo, 2012

025EAB97-35D1-548B-855C-36AF2391258A

https://www.marinespecies.org/aphia.php?p=taxdetails&id=1583931

##### Materials

- **Type status:** Paratype. **Occurrence:** catalogNumber: UANL 8156; recordedBy: Jesús A. de León-González; individualCount: 1; occurrenceID: 07A56641-4211-53B3-89F4-EA07677ACACA; **Taxon:** kingdom: Animalia; phylum: Annelida; class: Polychaeta; order: Terebellidae; family: Flabelligeridae; genus: Trophoniella; **Location:** continent: North America; waterBody: Gulf of California; country: Mexico; countryCode: MX; stateProvince: Sinaloa; municipality: Mazatlán; locality: Punta Cerritos; maximumDepthInMeters: 1; decimalLatitude: 23.308983; decimalLongitude: -106.493481; **Event:** eventDate: 02IV2004; habitat: Intertidal, mixed shore; **Record Level:** institutionCode: UANL; collectionCode: NL-INV-0002-05-09
- **Type status:** Paratype. **Occurrence:** catalogNumber: UANL 8157; recordedBy: María E. García-Garza | Jesús A. de León-González; individualCount: 6; occurrenceID: E7CDB63E-8A90-5692-BD98-9D756B1BB545; **Taxon:** kingdom: Animalia; phylum: Annelida; class: Polychaeta; order: Terebellidae; family: Flabelligeridae; genus: Trophoniella; **Location:** continent: North America; waterBody: Gulf of California; country: Mexico; countryCode: MX; stateProvince: Sinaloa; municipality: Mazatlán; locality: Punta Cerritos; maximumDepthInMeters: 1; decimalLatitude: 23.308983; decimalLongitude: -106.493481; **Event:** eventDate: 03III2006; habitat: Intertidal, mixed shore; **Record Level:** institutionCode: UANL; collectionCode: NL-INV-0002-05-09

#### Clymenura scutata

Tovar-Hernández & Yáñez-Rivera in Yáñez-Rivera et al. 2020

EA0B23C6-89FB-529F-A319-7BE212AB5B3D

https://www.marinespecies.org/aphia.php?p=taxdetails&id=1470587

##### Materials

- **Type status:** Paratype. **Occurrence:** catalogNumber: UANL 8144; recordedBy: Beatriz Yáñez-Rivera; individualCount: 3; occurrenceID: BB77B99C-5BD3-55E0-B6E2-4CE0A8198C2B; **Taxon:** kingdom: Animalia; phylum: Annelida; class: Polychaeta; order: Scolecida; family: Maldanidae; genus: Clymenura; **Location:** continent: North America; waterBody: Tropical Eastern Pacific; country: Mexico; countryCode: MX; stateProvince: Jalisco; municipality: Bahía de Chámela; locality: Isla Cocinas; maximumDepthInMeters: 7; decimalLatitude: 19.549167; decimalLongitude: -105.105556; **Event:** eventDate: 27VI2013; habitat: On rock; **Record Level:** institutionCode: UANL; collectionCode: NL-INV-0002-05-09

#### Struwela camposi

Salazar-Vallejo, de León-González & Carrera-Parra, 2019

D3472227-820B-51E2-821B-2DBBEE51065F

https://www.marinespecies.org/aphia.php?p=taxdetails&id=1467977

##### Materials

- **Type status:** Holotype. **Occurrence:** catalogNumber: UANL 8126; recordedBy: Ernesto Campos; occurrenceID: AE4F6A40-662B-535D-B6B6-FBD7A47BDE86; **Taxon:** kingdom: Animalia; phylum: Annelida; class: Polychaeta; order: Phyllodocida; family: Microphthalmidae; genus: Struwela; **Location:** continent: North America; waterBody: Temperate Northern Pacific; country: Mexico; countryCode: MX; stateProvince: Baja California; municipality: San Felipe; locality: Punta Estrella; decimalLatitude: 30.912083; decimalLongitude: -114.712222; **Event:** eventDate: September 1995; habitat: Intertidal on Encope grandis; **Record Level:** institutionCode: UANL; collectionCode: NL-INV-0002-05-09
- **Type status:** Paratype. **Occurrence:** catalogNumber: UANL 8127; recordedBy: Ernesto Campos; individualCount: 9; occurrenceID: 213ED6DA-9BBF-572D-A68F-BDB003306FCF; **Taxon:** kingdom: Animalia; phylum: Annelida; class: Polychaeta; order: Phyllodocida; family: Microphthalmidae; genus: Struwela; **Location:** continent: North America; waterBody: Temperate Northern Pacific; country: Mexico; countryCode: MX; stateProvince: Baja California; municipality: San Felipe; locality: Punta Estrella; decimalLatitude: 30.912083; decimalLongitude: -114.712222; **Event:** eventDate: September 1995; habitat: Intertidal on Lanthonia grantii; **Record Level:** institutionCode: UANL; collectionCode: NL-INV-0002-05-09

#### Neanthes philippinensis

de León-González & Salazar-Vallejo, 2003

538ACDA8-992B-5EDB-A535-A4FD93796426

https://www.marinespecies.org/aphia.php?p=taxdetails&id=329467

##### Materials

- **Type status:** Paratype. **Occurrence:** catalogNumber: UANL 5097; recordedBy: Scientific staff of MUSORSTROM 3; individualCount: 3; occurrenceID: 89448A29-26D7-5C2E-BF82-C175A5386371; **Taxon:** kingdom: Animalia; phylum: Annelida; class: Polychaeta; order: Phyllodocida; family: Nereididae; genus: Neanthes; **Location:** continent: Asia; waterBody: Indian Ocean; country: Phillipines; countryCode: PH; maximumDepthInMeters: 25; decimalLatitude: 11.783333; decimalLongitude: 123.033333; **Event:** eventDate: 23VI1985; **Record Level:** institutionCode: UANL; collectionCode: NL-INV-0002-05-09

#### Nereis baolingi

de León González & Solís-Weiss, 2000

1A1751E7-9626-564E-8D8B-46FCBEA5BC74

https://www.marinespecies.org/aphia.php?p=taxdetails&id=329610

##### Materials

- **Type status:** Paratype. **Occurrence:** catalogNumber: UANL 3982; recordedBy: Scientific staff of R/V VELERO IV; individualCount: 1; occurrenceID: A43787F4-EC4D-59D0-8E41-73EF19B44B59; **Taxon:** kingdom: Animalia; phylum: Annelida; class: Polychaeta; order: Phyllodocida; family: Nereididae; genus: Nereis; **Location:** continent: North America; waterBody: Temperate Northern Pacific; country: Mexico; countryCode: MX; stateProvince: Baja California Sur; locality: Punta Eugenia; decimalLatitude: 27.833333; decimalLongitude: -114.858333; **Event:** eventDate: 1951; habitat: On rock; **Record Level:** institutionCode: UANL; collectionCode: NL-INV-0002-05-09

#### Nereis casoae

de León-González & Solís-Weiss, 2001

C78845E0-794F-5B08-B1AC-C678A32804F6

https://www.marinespecies.org/aphia.php?p=taxdetails&id=329619

##### Materials

- **Type status:** Holotype. **Occurrence:** catalogNumber: UANL 4832; recordedBy: María E. Caso; occurrenceID: 875CBDE6-70B8-5B2A-9E7D-72C94DBB0E17; **Taxon:** kingdom: Animalia; phylum: Annelida; class: Polychaeta; order: Phyllodocida; family: Nereididae; genus: Nereis; **Location:** continent: North America; waterBody: Gulf of California; country: Mexico; countryCode: MX; stateProvince: Sinaloa; municipality: Mazatlán; locality: Isla Chivos; maximumDepthInMeters: 61; decimalLatitude: 23.182153; decimalLongitude: -106.411764; **Event:** eventDate: 09V1979; **Record Level:** institutionCode: UANL; collectionCode: NL-INV-0002-05-09
- **Type status:** Paratype. **Occurrence:** catalogNumber: UANL 4833; recordedBy: María E. Caso; individualCount: 6; occurrenceID: A2CAAF63-7806-598E-A92D-C8BBA0C57469; **Taxon:** kingdom: Animalia; phylum: Annelida; class: Polychaeta; order: Phyllodocida; family: Nereididae; genus: Nereis; **Location:** continent: North America; waterBody: Gulf of California; country: Mexico; countryCode: MX; stateProvince: Sinaloa; municipality: Mazatlán; locality: Isla Chivos; maximumDepthInMeters: 61; decimalLatitude: 23.182153; decimalLongitude: -106.411764; **Event:** eventDate: 09V1979; **Record Level:** institutionCode: UANL; collectionCode: NL-INV-0002-05-09

#### Nereis fauchaldi

de León-González & Díaz-Castañeda, 1998

1C80B8D1-D6F0-5482-84AD-F36BD8478513

https://www.marinespecies.org/aphia.php?p=taxdetails&id=329644

##### Materials

- **Type status:** Holotype. **Occurrence:** catalogNumber: UANL 3945; recordedBy: Victoria Díaz Castañeda; occurrenceID: 2E4B0789-990F-5C89-93BA-B90BCA589DD9; **Taxon:** kingdom: Animalia; phylum: Annelida; class: Polychaeta; order: Phyllodocida; family: Nereididae; genus: Nereis; **Location:** continent: North America; waterBody: Temperate Northern Pacific; country: Mexico; countryCode: MX; stateProvince: Baja California; locality: Todos Santos Bay; maximumDepthInMeters: 210; decimalLatitude: 31.783333; decimalLongitude: -116.733333; **Event:** eventDate: 11X1994; **Record Level:** institutionCode: UANL; collectionCode: NL-INV-0002-05-09

#### Nereis imajimai

de León González & Díaz-Castañeda, 1998

349EB556-6D95-59A2-9E3F-4651F902E121

https://www.marinespecies.org/aphia.php?p=taxdetails&id=329665

##### Materials

- **Type status:** Holotype. **Occurrence:** catalogNumber: UANL 3948; recordedBy: Victoria Díaz Castañeda; occurrenceID: 1A830131-1F4F-51CB-B873-5A123CDA4AD5; **Taxon:** kingdom: Animalia; phylum: Annelida; class: Polychaeta; order: Phyllodocida; family: Nereididae; genus: Nereis; **Location:** continent: North America; waterBody: Temperate Northern Pacific; country: Mexico; countryCode: MX; stateProvince: Baja California; locality: Todos Santos Bay; maximumDepthInMeters: 210; decimalLatitude: 31.783333; decimalLongitude: -116.733333; **Event:** eventDate: 22X1994; **Record Level:** institutionCode: UANL; collectionCode: NL-INV-0002-05-09

#### Nereis inflata

de León González & Solís-Weiss, 2001

142A3A04-D6B3-522A-9C77-742AC678246F

https://www.marinespecies.org/aphia.php?p=taxdetails&id=329668

##### Materials

- **Type status:** Paratype. **Occurrence:** catalogNumber: UANL 4835; recordedBy: Jesús A. de León-González; individualCount: 5; occurrenceID: 40AB2932-9B3F-5ABD-96F8-2A2212DBCFF5; **Taxon:** kingdom: Animalia; phylum: Annelida; class: Polychaeta; order: Phyllodocida; family: Nereididae; genus: Nereis; **Location:** continent: North America; waterBody: Temperate Northern Pacific; country: Mexico; countryCode: MX; stateProvince: Baja California Sur; locality: Punta San Juanico; maximumDepthInMeters: 30; decimalLatitude: 26.216667; decimalLongitude: -112.533333; **Event:** eventDate: 28II1989; **Record Level:** institutionCode: UANL; collectionCode: NL-INV-0002-05-09

#### Laeonereis watsoni

de León-González, Méndez & Navedo, 2018

B4DA750C-EEFA-5EE5-BF01-83D477AF9703

https://www.marinespecies.org/aphia.php?p=taxdetails&id=994381

##### Materials

- **Type status:** Holotype. **Occurrence:** catalogNumber: UANL 7843; recordedBy: Juan Navedo; occurrenceID: A4E21AF2-A11B-5044-82F0-99C073CD1A98; **Taxon:** kingdom: Animalia; phylum: Annelida; class: Polychaeta; order: Phyllodocida; family: Nereididae; genus: Laeonereis; **Location:** continent: North America; waterBody: Gulf of California; country: Mexico; countryCode: MX; stateProvince: Sinaloa; municipality: Mazatlán; locality: Estero de Urías; maximumDepthInMeters: 1; decimalLatitude: 23.151389; decimalLongitude: -106.289722; **Event:** eventDate: 05X2014; habitat: On shrimp ponds; **Record Level:** institutionCode: UANL; collectionCode: NL-INV-0002-05-09
- **Type status:** Paratype. **Occurrence:** catalogNumber: UANL 7844; recordedBy: Juan Navedo; individualCount: 5; occurrenceID: 0B719D8B-B081-5EE2-AEA5-E528EAB7A411; **Taxon:** kingdom: Animalia; phylum: Annelida; class: Polychaeta; order: Phyllodocida; family: Nereididae; genus: Laeonereis; **Location:** continent: North America; waterBody: Gulf of California; country: Mexico; countryCode: MX; stateProvince: Sinaloa; municipality: Mazatlán; locality: Estero de Urías; maximumDepthInMeters: 1; decimalLatitude: 23.151389; decimalLongitude: -106.289722; **Event:** eventDate: 05X2014; habitat: On shrimp ponds; **Record Level:** institutionCode: UANL; collectionCode: NL-INV-0002-05-09

#### Nicon orensanzi

de León-González & Trovant, 2013

6AF6F5B7-63C2-5A7C-A996-D5E32FC7AE16

https://www.marinespecies.org/aphia.php?p=taxdetails&id=836346

##### Materials

- **Type status:** Paratype. **Occurrence:** catalogNumber: UANL 7840; recordedBy: Berenice Trovant | Santiago Tineo; individualCount: 1; occurrenceID: EEB80A55-C9FB-5F1A-AE39-5C41FA8C746B; **Taxon:** kingdom: Animalia; phylum: Annelida; class: Polychaeta; order: Phyllodocida; family: Nereididae; genus: Nicon; **Location:** continent: South America; waterBody: Tropical Eastern Pacific; country: Ecuador; countryCode: ECU; stateProvince: Esmeraldas; locality: Bunche beach; decimalLatitude: 0.65055; decimalLongitude: -80.065281; **Event:** eventDate: 02III2009; habitat: intertidal; **Record Level:** institutionCode: UANL; collectionCode: NL-INV-0002-05-09

#### Nicon pettiboneae

de León-González & Salazar-Vallejo, 2003

505D3254-2952-5949-A999-A1956D501F22

https://www.marinespecies.org/aphia.php?p=taxdetails&id=329819

##### Materials

- **Type status:** Paratype. **Occurrence:** catalogNumber: UANL 5081; recordedBy: Scientific staff of CALSUB; individualCount: 1; occurrenceID: 5CCCF5EA-20DE-5940-A532-87B6BF595F6C; **Taxon:** kingdom: Animalia; phylum: Annelida; class: Polychaeta; order: Phyllodocida; family: Nereididae; genus: Nicon; **Location:** continent: Oceania; waterBody: Indian Ocean; country: New Caledonia; countryCode: NC; stateProvince: Exclusive Economic Zone; municipality: Loyalty islands; locality: Lifou and Santal; maximumDepthInMeters: 588; decimalLatitude: -20.883333; decimalLongitude: 167.05; **Event:** eventDate: 27II1989; **Record Level:** institutionCode: UANL; collectionCode: NL-INV-0002-05-09

#### Perinereis rookeri

de León-González & Goethel, 2013

3A4822AA-9F07-536D-9874-D13A6D5DD336

https://www.marinespecies.org/aphia.php?p=taxdetails&id=732744

##### Materials

- **Type status:** Holotype. **Occurrence:** catalogNumber: UANL 7841; occurrenceID: B896606D-409C-51B6-85C0-3420DF33E7DB; **Taxon:** kingdom: Animalia; phylum: Annelida; class: Polychaeta; order: Phyllodocida; family: Nereididae; genus: Perinereis; **Location:** continent: North America; waterBody: Caribbean Sea; country: United States of America; countryCode: USA; stateProvince: Florida; county: Naples; locality: Rookery Bay; decimalLatitude: 26.009333; decimalLongitude: -81.748333; **Event:** eventDate: 22XII2010; **Record Level:** institutionCode: UANL; collectionCode: NL-INV-0002-05-09
- **Type status:** Paratype. **Occurrence:** catalogNumber: UANL 7842; individualCount: 1; occurrenceID: 4C1A9DC6-2FC7-58AA-B12F-C95D2722D5AF; **Taxon:** kingdom: Animalia; phylum: Annelida; class: Polychaeta; order: Phyllodocida; family: Nereididae; genus: Perinereis; **Location:** continent: North America; waterBody: Caribbean Sea; country: United States of America; countryCode: USA; stateProvince: Florida; county: Naples; locality: Rookery Bay; decimalLatitude: 26.009333; decimalLongitude: -81.748333; **Event:** eventDate: 21XII2010; **Record Level:** institutionCode: UANL; collectionCode: NL-INV-0002-05-09

#### Platynereis hutchingsae

de León-González, Solís-Weiss & Valdez-Rocha, 2001

699B0F22-D04C-5301-8542-AA2BCC10D350

https://www.marinespecies.org/aphia.php?p=taxdetails&id=330806

##### Materials

- **Type status:** Holotype. **Occurrence:** catalogNumber: UANL 4281; recordedBy: Vivianne Solís-Weiss; occurrenceID: E91FE1D4-2CCE-5D97-B1B6-61A7B22CC730; **Taxon:** kingdom: Animalia; phylum: Annelida; class: Polychaeta; order: Phyllodocida; family: Nereididae; genus: Platynereis; **Location:** continent: North America; waterBody: Gulf of Mexico; country: Mexico; countryCode: MX; stateProvince: Campeche; municipality: Laguna de Términos; locality: San Julián; decimalLatitude: 18.758042; decimalLongitude: -91.493747; **Event:** eventDate: 03III1984; **Record Level:** institutionCode: UANL; collectionCode: NL-INV-0002-05-09
- **Type status:** Paratype. **Occurrence:** catalogNumber: UANL 4282; recordedBy: Vivianne Solís-Weiss; individualCount: 1; occurrenceID: B00BE876-DB48-5AE8-98F6-0BFBE179F1F0; **Taxon:** kingdom: Animalia; phylum: Annelida; class: Polychaeta; order: Phyllodocida; family: Nereididae; genus: Platynereis; **Location:** continent: North America; waterBody: Gulf of Mexico; country: Mexico; countryCode: MX; stateProvince: Campeche; municipality: Laguna de Términos; locality: San Julián; decimalLatitude: 18.758042; decimalLongitude: -91.493747; **Event:** eventDate: 03III1984; **Record Level:** institutionCode: UANL; collectionCode: NL-INV-0002-05-09

#### Platynereis mucronata

de León-González, Solís-Weiss & Valdez-Rocha, 2001

799650F6-388E-5C84-99C0-D699963206C5

https://www.marinespecies.org/aphia.php?p=taxdetails&id=330809

##### Materials

- **Type status:** Holotype. **Occurrence:** catalogNumber: UANL 3975; recordedBy: Jesús A. de León-González; occurrenceID: 34137F49-C2EF-5AC2-988E-EB05E1051F05; **Taxon:** kingdom: Animalia; phylum: Annelida; class: Polychaeta; order: Phyllodocida; family: Nereididae; genus: Platynereis; **Location:** continent: North America; waterBody: Gulf of Mexico; country: Mexico; countryCode: MX; stateProvince: Tamaulipas; locality: La Pesca; decimalLatitude: 23.771528; decimalLongitude: -97.73175000000001; **Event:** eventDate: 12IV1991; **Record Level:** institutionCode: UANL; collectionCode: NL-INV-0002-05-09

#### Rullierinereis fauchaldi

de León-González & Solís-Weiss, 2000

26500C88-6384-5DC7-B0AA-BC7BD876BBEC

https://www.marinespecies.org/aphia.php?p=taxdetails&id=331534

##### Materials

- **Type status:** Paratype. **Occurrence:** catalogNumber: UANL 3983; recordedBy: Scientific staff of VELERO IV; individualCount: 1; occurrenceID: BC63A4DB-B6A1-5FE3-9003-B8E1737A0163; **Taxon:** kingdom: Animalia; phylum: Annelida; class: Polychaeta; order: Phyllodocida; family: Nereididae; genus: Rulliernereis; **Location:** continent: North America; waterBody: Temperate Northern Pacific; country: Mexico; countryCode: MX; stateProvince: Baja California; locality: San Cristóbal Bay; decimalLatitude: 27.343889; decimalLongitude: -114.731944; **Event:** eventDate: 22III1959; **Record Level:** institutionCode: UANL; collectionCode: NL-INV-0002-05-09

#### Stenoninereis tecolutlensis

de León-González & Solís-Weiss, 1997

F34465D5-414C-5332-97D0-2CFC8E311917

https://www.marinespecies.org/aphia.php?p=taxdetails&id=421050

##### Materials

- **Type status:** Paratype. **Occurrence:** catalogNumber: UANL 3980; recordedBy: Jesús A. de León-González; individualCount: 1; occurrenceID: 4F24D13A-A34D-5736-9DFE-2DB4E5CA2CA1; **Taxon:** kingdom: Animalia; phylum: Annelida; class: Polychaeta; order: Phyllodocida; family: Nereididae; genus: Stenoninereis; **Location:** continent: North America; waterBody: Gulf of Mexico; country: Mexico; countryCode: MX; stateProvince: Veracruz; municipality: Tecolutla; locality: Estero Larios; decimalLatitude: 20.468269; decimalLongitude: -97.007972; **Event:** eventDate: 19XI1994; **Record Level:** institutionCode: UANL; collectionCode: NL-INV-0002-05-09

#### Websterinereis pettiboneae

de León-González & Balart, 2016

28866CBB-87FB-52DF-8B83-9F68B3FD7D69

https://www.marinespecies.org/aphia.php?p=taxdetails&id=884154

##### Materials

- **Type status:** Holotype. **Occurrence:** catalogNumber: UANL 7845; recordedBy: Jesús A. de León-González; occurrenceID: 386C8F31-FE42-53DD-A0C0-4963E86BD456; **Taxon:** kingdom: Animalia; phylum: Annelida; class: Polychaeta; order: Phyllodocida; family: Nereididae; genus: Websterinereis; **Location:** continent: North America; waterBody: Gulf of California; country: Mexico; countryCode: MX; stateProvince: Baja California Sur; municipality: La Paz Bay; locality: San Lorenzo Channel; decimalLatitude: 24.3865; decimalLongitude: -110.315417; **Event:** eventDate: July 2006; **Record Level:** institutionCode: UANL; collectionCode: NL-INV-0002-05-09

#### Australonuphis paxtonae

de León-González, Cornejo-Rodríguez & Degraer, 2008

F85BE0C8-D404-561A-90AF-81AF79A52DA6

https://www.marinespecies.org/aphia.php?p=taxdetails&id=731086

##### Materials

- **Type status:** Holotype. **Occurrence:** catalogNumber: UANL 6488; recordedBy: María H. Cornejo-Rodríguez; occurrenceID: 17F90BF7-4DEB-5E8D-BB0B-36B3D682CBDC; **Taxon:** kingdom: Animalia; phylum: Annelida; class: Polychaeta; order: Eunicida; family: Onuphidae; genus: Australonuphis; **Location:** continent: South America; waterBody: Tropical Eastern Pacific; country: Ecuador; countryCode: ECU; locality: Santa Elena Bay; decimalLatitude: -1.941667; decimalLongitude: -80.72499; **Event:** eventDate: 15XI2006; habitat: intertidal; **Record Level:** institutionCode: UANL; collectionCode: NL-INV-0002-05-09
- **Type status:** Paratype. **Occurrence:** catalogNumber: UANL 6489; recordedBy: María H. Cornejo-Rodríguez; individualCount: 1; occurrenceID: AE28BB68-6C68-525D-878E-619D2C108F1B; **Taxon:** kingdom: Animalia; phylum: Annelida; class: Polychaeta; order: Eunicida; family: Onuphidae; genus: Australonuphis; **Location:** continent: South America; waterBody: Tropical Eastern Pacific; country: Ecuador; countryCode: ECU; locality: Santa Elena Bay; decimalLatitude: -1.941667; decimalLongitude: -80.72499; **Event:** eventDate: 15XI2006; habitat: intertidal; **Record Level:** institutionCode: UANL; collectionCode: NL-INV-0002-05-09

#### Kinbergonuphis kristiani

de León-González, Rivera & Romero, 2004

C42EBEFC-5782-53EC-B704-5AE1403C80E0

https://www.marinespecies.org/aphia.php?p=taxdetails&id=328550

##### Materials

- **Type status:** Holotype. **Occurrence:** catalogNumber: UANL 5490; occurrenceID: A6CADC53-F2F8-5F09-82B9-20F1BA8735A5; **Taxon:** kingdom: Animalia; phylum: Annelida; class: Polychaeta; order: Eunicida; family: Onuphidae; genus: Kinbergonuphis; **Location:** continent: North America; waterBody: Tropical Eastern Pacific; country: El Salvador; countryCode: SV; locality: Off La Libertad; decimalLatitude: 13.385; decimalLongitude: -89.528333; **Event:** eventDate: 16III2001; **Record Level:** institutionCode: UANL; collectionCode: NL-INV-0002-05-09

#### Paradiopatra barrazai

de León-González, Rivera & Romero, 2004

BED3C3C0-73E5-5881-B44E-A186CEB19DC0

https://www.marinespecies.org/aphia.php?p=taxdetails&id=330272

##### Materials

- **Type status:** Holotype. **Occurrence:** catalogNumber: UANL 5491; occurrenceID: C7370037-D5D6-5513-A15B-7302AF476967; **Taxon:** kingdom: Animalia; phylum: Annelida; class: Polychaeta; order: Eunicida; family: Onuphidae; genus: Paradiopatra; **Location:** continent: North America; waterBody: Tropical Eastern Pacific; country: El Salvador; countryCode: SV; locality: Jalisquillo Bay; maximumDepthInMeters: 20; decimalLatitude: 13.065333; decimalLongitude: -88.667; **Event:** eventDate: 17III2001; **Record Level:** institutionCode: UANL; collectionCode: NL-INV-0002-05-09

#### Leitoscoloplos bajacaliforniensis

de León-González & Rodríguez, 1996

ADD5ABD8-E0AF-5D37-B1EB-99B76D77E0BE

https://www.marinespecies.org/aphia.php?p=taxdetails&id=849316

##### Materials

- **Type status:** Holotype. **Occurrence:** catalogNumber: UANL 0691; occurrenceID: D87CDE55-2CDE-5C10-BFD6-2C472E15D5D7; **Taxon:** kingdom: Animalia; phylum: Annelida; class: Polychaeta; order: Scolecida; family: Orbiniidae; genus: Leitoscoloplos; **Location:** continent: North America; waterBody: Temperate Northern Pacific; country: Mexico; countryCode: MX; stateProvince: Baja California; locality: Western coast of Baja Peninsula; maximumDepthInMeters: 103; decimalLatitude: 28.168333; decimalLongitude: -114.735; **Event:** eventDate: 05V1989; **Record Level:** institutionCode: UANL; collectionCode: NL-INV-0002-05-09

#### Galathowenia kirkegaardi

de León-González & Sánchez-Hernández, 2012

A6693C3A-7E4F-5894-8094-80B2FD53BE5D

https://www.marinespecies.org/aphia.php?p=taxdetails&id=598783

##### Materials

- **Type status:** Holotype. **Occurrence:** catalogNumber: UANL 7425; recordedBy: Jesús A. de León-González; occurrenceID: F974092D-635E-5D84-8D5E-D8947EADA715; **Taxon:** kingdom: Animalia; phylum: Annelida; class: Polychaeta; family: Oweniidae; genus: Galathowenia; **Location:** continent: North America; waterBody: Gulf of Mexico; country: Mexico; countryCode: MX; stateProvince: Veracruz; locality: Tamiahua; maximumDepthInMeters: 2.1; decimalLatitude: 21.646389; decimalLongitude: -97.520556; **Event:** eventDate: 23II2002; **Record Level:** institutionCode: UANL; collectionCode: NL-INV-0002-05-09
- **Type status:** Paratype. **Occurrence:** catalogNumber: UANL 7426; recordedBy: Jesús A. de León-González; individualCount: 10; occurrenceID: A29DECA6-7AA4-5E16-BC50-6972FC2C8032; **Taxon:** kingdom: Animalia; phylum: Annelida; class: Polychaeta; family: Oweniidae; genus: Galathowenia; **Location:** continent: North America; waterBody: Gulf of Mexico; country: Mexico; countryCode: MX; stateProvince: Veracruz; locality: Tamiahua; maximumDepthInMeters: 2.1; decimalLatitude: 21.646389; decimalLongitude: -97.520556; **Event:** eventDate: 23II2003; **Record Level:** institutionCode: UANL; collectionCode: NL-INV-0002-05-09

#### Aricidea (Aricidea) petacalcoensis

de León-González, Hernández-Guevara & Rodríguez-Valencia, 2006

C57198EC-D5E0-59E5-A93F-3AD1C280739B

https://www.marinespecies.org/aphia.php?p=taxdetails&id=731360

##### Materials

- **Type status:** Holotype. **Occurrence:** catalogNumber: UANL 6324; recordedBy: Edgar Amador-Silva | Juan J. Ramírez-Rosa; occurrenceID: 0FEE6AC3-364A-5981-A5F5-778E4315AFE9; **Taxon:** kingdom: Animalia; phylum: Annelida; class: Polychaeta; order: Scolecida; family: Paraonidae; genus: Aricidea; **Location:** continent: North America; waterBody: Tropical Eastern Pacific; country: Mexico; countryCode: MX; stateProvince: Guerrero; locality: Petacalco Bay; maximumDepthInMeters: 11; decimalLatitude: 17.911389; decimalLongitude: -102.043333; **Event:** eventDate: 09II1994; **Record Level:** institutionCode: UANL; collectionCode: NL-INV-0002-05-09
- **Type status:** Paratype. **Occurrence:** catalogNumber: UANL 6331; recordedBy: Edgar Amador-Silva | Juan J. Ramírez-Rosa; individualCount: 3; occurrenceID: FC800013-6D12-579E-A424-CED198CED9F2; **Taxon:** kingdom: Animalia; phylum: Annelida; class: Polychaeta; order: Scolecida; family: Paraonidae; genus: Aricidea; subgenus: Aricidea; **Location:** continent: North America; waterBody: Tropical Eastern Pacific; country: Mexico; countryCode: MX; stateProvince: Guerrero; locality: Petacalco Bay; maximumDepthInMeters: 11; decimalLatitude: 17.911389; decimalLongitude: -102.043333; **Event:** eventDate: 07X1993; **Record Level:** institutionCode: UANL; collectionCode: NL-INV-0002-05-09

#### Paradoneis magdalenaensis

(de León-González, Hernández-Guevara & Rodríguez-Valencia, 2006)

50D33102-CB81-5CFE-8F54-C11A5DE9AAE0

https://www.marinespecies.org/aphia.php?p=taxdetails&id=389905

##### Materials

- **Type status:** Holotype. **Occurrence:** catalogNumber: UANL 6325; recordedBy: Victoria Díaz Castañeda; occurrenceID: 6D64CFDE-B17E-5594-BE9F-5FA4826B73BF; **Taxon:** kingdom: Animalia; phylum: Annelida; class: Polychaeta; order: Scolecida; family: Paraonidae; genus: Cirrophorus; **Location:** continent: North America; waterBody: Temperate Northern Pacific; country: Mexico; countryCode: MX; stateProvince: Baja California Sur; locality: Bahía Magdalena; maximumDepthInMeters: 74; decimalLatitude: 24.519444; decimalLongitude: -112.086111; **Event:** eventDate: 06XII1996; **Record Level:** institutionCode: UANL; collectionCode: NL-INV-0002-05-09
- **Type status:** Paratype. **Occurrence:** catalogNumber: UANL 6326; recordedBy: Victoria Díaz Castañeda; individualCount: 2; occurrenceID: 09D69921-F4C2-520E-83EB-F19755223A13; **Taxon:** kingdom: Animalia; phylum: Annelida; class: Polychaeta; order: Scolecida; family: Paraonidae; genus: Cirrophorus; **Location:** continent: North America; waterBody: Temperate Northern Pacific; country: Mexico; countryCode: MX; stateProvince: Baja California Sur; locality: Bahía Magdalena; maximumDepthInMeters: 74; decimalLatitude: 24.519444; decimalLongitude: -112.086111; **Event:** eventDate: 06XII1996; **Record Level:** institutionCode: UANL; collectionCode: NL-INV-0002-05-09

#### Paradoneis strelzovi

de León-González & Díaz-Castañeda, 2011

995C50DC-374E-505F-82E9-41247B536734

https://www.marinespecies.org/aphia.php?p=taxdetails&id=598828

##### Materials

- **Type status:** Holotype. **Occurrence:** catalogNumber: UANL 6333; recordedBy: Victoria Díaz Castañeda; occurrenceID: 2503AF80-43E5-5C6A-97D1-A265F4557660; **Taxon:** kingdom: Animalia; phylum: Annelida; class: Polychaeta; order: Scolecida; family: Paraonidae; genus: Paradoneis; **Location:** continent: North America; waterBody: Temperate Northern Pacific; country: Mexico; countryCode: MX; stateProvince: Baja California; municipality: Ensenada; locality: Salsipuedes Bay; maximumDepthInMeters: 49; decimalLatitude: 31.963611; decimalLongitude: -116.809167; **Event:** eventDate: 04III2003; **Record Level:** institutionCode: UANL; collectionCode: NL-INV-0002-05-09
- **Type status:** Paratype. **Occurrence:** catalogNumber: UANL 6334; recordedBy: Victoria Díaz Castañeda; individualCount: 10; occurrenceID: A9357D80-EB3A-5BF0-85F5-E95AF8D150A9; **Taxon:** kingdom: Animalia; phylum: Annelida; class: Polychaeta; order: Scolecida; family: Paraonidae; genus: Paradoneis; **Location:** continent: North America; waterBody: Temperate Northern Pacific; country: Mexico; countryCode: MX; stateProvince: Baja California; municipality: Ensenada; locality: Salsipuedes Bay; maximumDepthInMeters: 49; decimalLatitude: 31.963611; decimalLongitude: -116.809167; **Event:** eventDate: 04III2004; **Record Level:** institutionCode: UANL; collectionCode: NL-INV-0002-05-09

#### Amphictene helenae

García-Garza & de León González, 2014

FE84E8F2-D52E-5105-B3DB-5F377D27CB7A

https://www.marinespecies.org/aphia.php?p=taxdetails&id=819663

##### Materials

- **Type status:** Holotype. **Occurrence:** catalogNumber: UANL 7824; recordedBy: María E. García-Garza | Julio H. Landín-Delgado; occurrenceID: 4CE209B2-40DB-5F7A-81BD-4B098D5B7554; **Taxon:** kingdom: Animalia; phylum: Annelida; class: Polychaeta; order: Scolecida; family: Pectinariidae; genus: Amphictene; **Location:** continent: North America; waterBody: Gulf of Mexico; country: Mexico; countryCode: MX; stateProvince: Campeche; municipality: Ciudad del Carmen; locality: Bahamintas Beach; maximumDepthInMeters: 0.5; decimalLatitude: 18.7; decimalLongitude: -91.683333; **Event:** eventDate: 03I2011; **Record Level:** institutionCode: UANL; collectionCode: NL-INV-0002-05-09

#### Pectinaria santii

Tovar-Hernández & de León-González, 2022

0922CA6B-E6C6-5A73-A875-9C2CB5E4C6EF

https://www.marinespecies.org/aphia.php?p=taxdetails&id=1608633

##### Materials

- **Type status:** Paratype. **Occurrence:** catalogNumber: UANL 8152; recordedBy: Santiago Hernández | María A. Tovar-Hernández; individualCount: 7; preparations: Whole animal (ETOH); occurrenceID: BB5B220A-5329-5651-A1D6-70F37468A55B; **Taxon:** kingdom: Animalia; phylum: Annelida; class: Polychaeta; order: Scolecida; family: Pectinariidae; genus: Pectinaria; **Location:** continent: North America; waterBody: Gulf of California; country: Mexico; countryCode: MX; stateProvince: Sinaloa; municipality: Ahome; locality: El Mavirí; maximumDepthInMeters: 0.2; decimalLatitude: 25.581944; decimalLongitude: -109.114722; **Event:** eventDate: 08IV2021; **Record Level:** institutionCode: UANL; collectionCode: NL-INV-0002-05-09

#### Synelmis emiliae

Salazar-Vallejo, 2003

F1E2AC52-EB71-58AF-B9C7-8EAD247AA616

https://www.marinespecies.org/aphia.php?p=taxdetails&id=332436

##### Materials

- **Type status:** Syntype. **Occurrence:** catalogNumber: UANL 0318; recordedBy: Sergio I. Salazar-Vallejo; individualCount: 1; occurrenceID: E6CBB2F9-DC2D-5578-9B85-1486DB6F8F96; **Taxon:** kingdom: Animalia; phylum: Annelida; class: Polychaeta; order: Phyllodocida; family: Pilargidae; genus: Synelmis; **Location:** continent: North America; waterBody: Gulf of California; country: Mexico; countryCode: MX; stateProvince: Baja California Sur; municipality: Concepción Bay; locality: Santispac; decimalLatitude: 26.768889; decimalLongitude: -111.897222; **Event:** eventDate: 18VII1985; habitat: intertidal; **Record Level:** institutionCode: UANL; collectionCode: NL-INV-0002-05-09

#### Acromegalomma schwindtae

Tovar-Hernández, de León-González & Bybee, 2017

02924F74-044A-57DF-9BF9-283D58F2B9EC

https://www.marinespecies.org/aphia.php?p=taxdetails&id=1006755

##### Materials

- **Type status:** Holotype. **Occurrence:** catalogNumber: UANL 8042; recordedBy: Evangelina Schwindt; occurrenceID: 202503DB-92E8-5C40-8F79-BF43EB0F12EE; **Taxon:** kingdom: Animalia; phylum: Annelida; class: Polychaeta; order: Sabellida; family: Sabellidae; genus: Acromegalomma; **Location:** continent: South America; waterBody: Patagonian Shelf; country: Argentina; countryCode: ARG; stateProvince: Provincia de Río Negro; locality: Puerto de San Antonio Este; maximumDepthInMeters: 9; decimalLatitude: -40.824994; decimalLongitude: -64.749878; **Event:** eventDate: 10X2005; habitat: Dock pilling fouling; **Record Level:** institutionCode: UANL; collectionCode: NL-INV-0002-05-09
- **Type status:** Paratype. **Occurrence:** catalogNumber: UANL 8043; recordedBy: Evangelina Schwindt; individualCount: 2; occurrenceID: 94A869EA-D663-55CC-80F9-80A7CA1C56AE; **Taxon:** kingdom: Animalia; phylum: Annelida; class: Polychaeta; order: Sabellida; family: Sabellidae; genus: Acromegalomma; **Location:** continent: South America; waterBody: Patagonian Shelf; country: Argentina; countryCode: ARG; stateProvince: Provincia de Río Negro; locality: Puerto de San Antonio Este; maximumDepthInMeters: 9; decimalLatitude: -40.824994; decimalLongitude: -64.749878; **Event:** eventDate: 10X2006; **Record Level:** institutionCode: UANL; collectionCode: NL-INV-0002-05-09

#### Chone orensanzi

Tovar-Hernández, de León-González & Bybee, 2017

542F65C5-8526-5131-A53B-F796397F2FDC

https://www.marinespecies.org/aphia.php?p=taxdetails&id=1006754

##### Materials

- **Type status:** Holotype. **Occurrence:** catalogNumber: UANL 8007; recordedBy: José M. Orensanz; occurrenceID: E8AB53CB-82FA-5528-AF23-F83548941CCC; **Taxon:** kingdom: Animalia; phylum: Annelida; class: Polychaeta; order: Sabellida; family: Sabellidae; genus: Chone; **Location:** continent: South America; waterBody: Patagonian Shelf; country: Argentina; countryCode: ARG; stateProvince: Golfo de San Matías; maximumDepthInMeters: 12.5; decimalLatitude: -40.95; decimalLongitude: -65.05; **Event:** eventDate: 17II1971; **Record Level:** institutionCode: UANL; collectionCode: NL-INV-0002-05-09
- **Type status:** Paratype. **Occurrence:** catalogNumber: UANL 8008; recordedBy: José M. Orensanz; individualCount: 38; occurrenceID: 4364E8BA-C255-58A4-B45C-9F337C033CF5; **Taxon:** kingdom: Animalia; phylum: Annelida; class: Polychaeta; order: Sabellida; family: Sabellidae; genus: Chone; **Location:** continent: South America; waterBody: Patagonian Shelf; country: Argentina; countryCode: ARG; stateProvince: Golfo de San Matías; maximumDepthInMeters: 12.5; decimalLatitude: -40.95; decimalLongitude: -65.05; **Event:** eventDate: 17II1971; **Record Level:** institutionCode: UANL; collectionCode: NL-INV-0002-05-09

#### Claviramus kyushuensis

Nishi, Tanaka & Tovar-Hernández, 2019

3CA6D4B1-7E43-5D80-AED3-C983D8F79E8B

https://www.marinespecies.org/aphia.php?p=taxdetails&id=1382722

##### Materials

- **Type status:** Paratype. **Occurrence:** catalogNumber: UANL 8130; recordedBy: Keisuke Mori; individualCount: 3; occurrenceID: 6803DCE0-75C5-5CB2-8C0C-88DE92C7AFD5; **Taxon:** kingdom: Animalia; phylum: Annelida; class: Polychaeta; order: Sabellida; family: Sabellidae; genus: Claviramus; **Location:** continent: Asia; waterBody: Temperate Northern Pacific; country: Japan; countryCode: JPN; stateProvince: Ariake Sound; locality: Kyushu; maximumDepthInMeters: 20; decimalLatitude: 32.517833; decimalLongitude: 130.23395; **Event:** eventDate: 07IX2005; **Record Level:** institutionCode: UANL; collectionCode: NL-INV-0002-05-09

#### Notaulax nigroincrustata

Tovar-Hernández, García-Garza & de León-González, 2020

885702AC-9545-5060-B79F-117DDCD3A619

https://www.marinespecies.org/aphia.php?p=taxdetails&id=1470583

##### Materials

- **Type status:** Holotype. **Occurrence:** catalogNumber: UANL 8138; recordedBy: José M. Aguilar-Camacho | Irving D. Ramírez-Santana; occurrenceID: 0D44456F-8F40-5FC0-BB7F-562BA828D5C8; **Taxon:** kingdom: Animalia; phylum: Annelida; class: Polychaeta; order: Sabellida; family: Sabellidae; genus: Notaulax; **Location:** continent: North America; waterBody: Gulf of California; country: Mexico; countryCode: MX; stateProvince: Baja California Sur; municipality: La Paz Bay; locality: Marina La Paz; maximumDepthInMeters: 1; decimalLatitude: 24.154933; decimalLongitude: -110.3261; **Event:** eventDate: 14VIII2011; habitat: Dock pilling fouling; **Record Level:** institutionCode: UANL; collectionCode: NL-INV-0002-05-09
- **Type status:** Paratype. **Occurrence:** catalogNumber: UANL 8139; recordedBy: José M. Aguilar-Camacho | Irving D. Ramírez-Santana; individualCount: 2; occurrenceID: 5A4C7411-CA87-57FF-90E5-AE89E42148AB; **Taxon:** kingdom: Animalia; phylum: Annelida; class: Polychaeta; order: Sabellida; family: Sabellidae; genus: Notaulax; **Location:** continent: North America; waterBody: Gulf of California; country: Mexico; countryCode: MX; stateProvince: Baja California Sur; municipality: La Paz Bay; locality: Marina La Paz; maximumDepthInMeters: 1; decimalLatitude: 24.154933; decimalLongitude: -110.3261; **Event:** eventDate: 14VIII2011; habitat: Dock pilling fouling; **Record Level:** institutionCode: UANL; collectionCode: NL-INV-0002-05-09
- **Type status:** Paratype. **Occurrence:** catalogNumber: UANL 8140; recordedBy: José M. Aguilar-Camacho | Irving D. Ramírez-Santana; individualCount: 3; occurrenceID: 353557BA-4A09-5D94-B8A8-5019BDF20566; **Taxon:** kingdom: Animalia; phylum: Annelida; class: Polychaeta; order: Sabellida; family: Sabellidae; genus: Notaulax; **Location:** continent: North America; waterBody: Gulf of California; country: Mexico; countryCode: MX; stateProvince: Baja California Sur; municipality: La Paz Bay; locality: Marina La Paz; maximumDepthInMeters: 1; decimalLatitude: 24.154933; decimalLongitude: -110.3261; **Event:** eventDate: 14VIII2011; habitat: Dock pilling fouling; **Record Level:** institutionCode: UANL; collectionCode: NL-INV-0002-05-09

#### Notaulax punctulata

Tovar-Hernández, García-Garza & de León-González, 2020

8985C73B-0093-5320-AFB0-C9377C92E27D

https://www.marinespecies.org/aphia.php?p=taxdetails&id=1470584

##### Materials

- **Type status:** Holotype. **Occurrence:** catalogNumber: UANL 8142; recordedBy: Tulio F. Villalobos-Guerrero; occurrenceID: AC2A1348-03F2-5DEE-ACCB-9472C54C0917; **Taxon:** kingdom: Animalia; phylum: Annelida; class: Polychaeta; order: Sabellida; family: Sabellidae; genus: Notaulax; **Location:** continent: North America; waterBody: Tropical Eastern Pacific; country: Mexico; countryCode: MX; stateProvince: Guerrero; municipality: Acapulco; locality: Playa Hornitos; maximumDepthInMeters: 3; decimalLatitude: 16.857336; decimalLongitude: -99.889083; **Event:** eventDate: 09XI2015; **Record Level:** institutionCode: UANL; collectionCode: NL-INV-0002-05-09
- **Type status:** Paratype. **Occurrence:** catalogNumber: UANL 8143; recordedBy: Tulio F. Villalobos-Guerrero; individualCount: 1; occurrenceID: C51C6CBA-443B-51A6-8485-8720C1EECA45; **Taxon:** kingdom: Animalia; phylum: Annelida; class: Polychaeta; order: Sabellida; family: Sabellidae; genus: Notaulax; **Location:** continent: North America; waterBody: Tropical Eastern Pacific; country: Mexico; countryCode: MX; stateProvince: Guerrero; municipality: Acapulco; locality: Playa Hornitos; maximumDepthInMeters: 3; decimalLatitude: 16.857336; decimalLongitude: -99.889083; **Event:** eventDate: 09XI2015; **Record Level:** institutionCode: UANL; collectionCode: NL-INV-0002-05-09

#### Notaulax salazari

Tovar-Hernández, de León-González & Bybee, 2017

59CB6884-1A46-50D1-8988-E1031058D178

https://www.marinespecies.org/aphia.php?p=taxdetails&id=1006762

##### Materials

- **Type status:** Holotype. **Occurrence:** catalogNumber: UANL 8060; recordedBy: Evangelina Schwindt; occurrenceID: 8A938CF5-C758-5955-B01E-E48AC41BA64D; **Taxon:** kingdom: Animalia; phylum: Annelida; class: Polychaeta; order: Sabellida; family: Sabellidae; genus: Notaulax; **Location:** continent: South America; waterBody: Patagonian Shelf; country: Argentina; countryCode: ARG; stateProvince: Provincia de Santa Cruz; locality: Puerto Deseado; decimalLatitude: -47.759208; decimalLongitude: -65.90495799999999; **Event:** eventDate: 18X2005; habitat: Fouling; **Record Level:** institutionCode: UANL; collectionCode: NL-INV-0002-05-09
- **Type status:** Paratype. **Occurrence:** catalogNumber: UANL 8061; recordedBy: Evangelina Schwindt; individualCount: 2; occurrenceID: 4BC52D0C-2C70-5052-B199-D8F4B382AC17; **Taxon:** kingdom: Animalia; phylum: Annelida; class: Polychaeta; order: Sabellida; family: Sabellidae; genus: Notaulax; **Location:** continent: South America; waterBody: Patagonian Shelf; country: Argentina; countryCode: ARG; stateProvince: Provincia de Santa Cruz; locality: Puerto Deseado; decimalLatitude: -47.759208; decimalLongitude: -65.90495799999999; **Event:** eventDate: 18X2005; habitat: Fouling; **Record Level:** institutionCode: UANL; collectionCode: NL-INV-0002-05-09

#### Parasabella yonowae

Tovar-Hernández, de León-González & Bybee, 2017

8CA1BF77-4631-5A8D-ABFA-D8BF4ADD233D

https://www.marinespecies.org/aphia.php?p=taxdetails&id=1006763

##### Materials

- **Type status:** Holotype. **Occurrence:** catalogNumber: UANL 8069; recordedBy: José M. Orensanz; occurrenceID: 0575AA29-A876-54A9-BDBF-F51919C95EF3; **Taxon:** kingdom: Animalia; phylum: Annelida; class: Polychaeta; order: Sabellida; family: Sabellidae; genus: Parasabella; **Location:** continent: South America; waterBody: Patagonian Shelf; country: Argentina; countryCode: ARG; stateProvince: Golfo de San José; locality: El Riacho; decimalLatitude: -42.4; decimalLongitude: -64.583333; **Event:** eventDate: t27881; habitat: Subtidal; **Record Level:** institutionCode: UANL; collectionCode: NL-INV-0002-05-09
- **Type status:** Paratype. **Occurrence:** catalogNumber: UANL 8070; recordedBy: José M. Orensanz; individualCount: 3; occurrenceID: 58C3100E-F80B-5C78-BB05-85E8D636F8B8; **Taxon:** kingdom: Animalia; phylum: Annelida; class: Polychaeta; order: Sabellida; family: Sabellidae; genus: Parasabella; **Location:** continent: South America; waterBody: Patagonian Shelf; country: Argentina; countryCode: ARG; stateProvince: Golfo de San José; locality: El Riacho; decimalLatitude: -42.4; decimalLongitude: -64.583333; **Event:** eventDate: t27881; habitat: Subtidal; **Record Level:** institutionCode: UANL; collectionCode: NL-INV-0002-05-09

#### Branchiosyllis riojai

Góngora-Garza, García-Garza & de León-González, 2011

AAA2970F-A357-5896-8B8E-328262907B44

https://www.marinespecies.org/aphia.php?p=taxdetails&id=598770

##### Materials

- **Type status:** Holotype. **Occurrence:** catalogNumber: UANL 6787; recordedBy: Jesús A. de León-González; occurrenceID: EE315EED-9A02-59E8-8C4F-A3562124EAFC; **Taxon:** kingdom: Animalia; phylum: Annelida; class: Polychaeta; order: Phyllodocida; family: Syllidae; genus: Branchiosyllis; **Location:** continent: North America; waterBody: Tropical Eastern Pacific; country: Mexico; countryCode: MX; stateProvince: Colima; municipality: Manzanillo; locality: Marina Dorada; maximumDepthInMeters: 1.5; decimalLatitude: 19.062544; decimalLongitude: -104.300178; **Event:** eventDate: 07VI2004; **Record Level:** institutionCode: UANL; collectionCode: NL-INV-0002-05-09

#### Branchiosyllis sanmartini

Góngora-Garza, García-Garza & de León-González, 2011

CA1FAE8F-2177-5682-A183-C2B119F9E78C

https://www.marinespecies.org/aphia.php?p=taxdetails&id=598769

##### Materials

- **Type status:** Holotype. **Occurrence:** catalogNumber: UANL 6785; recordedBy: Sergio I. Salazar-Vallejo; occurrenceID: 339BCFE7-122D-5FDA-949E-D97AA15EDDCF; **Taxon:** kingdom: Animalia; phylum: Annelida; class: Polychaeta; order: Phyllodocida; family: Syllidae; genus: Branchiosyllis; **Location:** continent: North America; waterBody: Gulf of California; country: Mexico; countryCode: MX; stateProvince: Baja California Sur; municipality: La Paz Bay; locality: Malecón; decimalLatitude: 24.158669; decimalLongitude: -110.319919; **Event:** eventDate: 04VIII2004; habitat: intertidal among algae attached to rocks; **Record Level:** institutionCode: UANL; collectionCode: NL-INV-0002-05-09
- **Type status:** Paratype. **Occurrence:** catalogNumber: UANL 6786; recordedBy: Sergio I. Salazar-Vallejo; individualCount: 5; occurrenceID: D7C1FF07-4128-5356-8B7D-E5433BF503A7; **Taxon:** kingdom: Animalia; phylum: Annelida; class: Polychaeta; order: Phyllodocida; family: Syllidae; genus: Branchiosyllis; **Location:** continent: North America; waterBody: Gulf of California; country: Mexico; countryCode: MX; stateProvince: Baja California Sur; municipality: La Paz Bay; locality: Malecón; decimalLatitude: 24.158669; decimalLongitude: -110.319919; **Event:** eventDate: 04VIII2004; habitat: intertidal among algae attached to rocks; **Record Level:** institutionCode: UANL; collectionCode: NL-INV-0002-05-09

#### Parasphaerosyllis irregulata

Góngora-Garza, Tovar-Hernández & de León-González, 2024

93B0B159-9C2C-56A1-A7B3-399EE43BB69C

##### Materials

- **Type status:** Holotype. **Occurrence:** catalogNumber: UANL 8158; recordedBy: Jesús A. de León-González; occurrenceID: A9099E0D-CD1C-5749-A916-F79204BF3DD9; **Taxon:** kingdom: Animalia; phylum: Annelida; class: Polychaeta; order: Phyllodocida; family: Syllidae; genus: Parasphaerosyllis; **Location:** continent: North America; waterBody: Gulf of California; country: Mexico; countryCode: MX; stateProvince: Baja California Sur; municipality: La Paz Bay; locality: San Lorenzo Channel; decimalLatitude: 24.3865; decimalLongitude: -110.315417; **Event:** eventDate: 05V2015; habitat: intertidal; **Record Level:** institutionCode: UANL; collectionCode: NL-INV-0002-05-09
- **Type status:** Paratype. **Occurrence:** catalogNumber: UANL 8159; recordedBy: Jesús A. de León-González; individualCount: 1; occurrenceID: 53DD1217-1759-54C9-9E40-0F760B251827; **Taxon:** kingdom: Animalia; phylum: Annelida; class: Polychaeta; order: Phyllodocida; family: Syllidae; genus: Parasphaerosyllis; **Location:** continent: North America; waterBody: Gulf of California; country: Mexico; countryCode: MX; stateProvince: Baja California Sur; municipality: La Paz Bay; locality: San Lorenzo Channel; decimalLatitude: 24.3865; decimalLongitude: -110.315417; **Event:** eventDate: 05V2015; habitat: intertidal; **Record Level:** institutionCode: UANL; collectionCode: NL-INV-0002-05-09
- **Type status:** Paratype. **Occurrence:** catalogNumber: UANL 8160; recordedBy: Jesús A. de León-González; individualCount: 2; occurrenceID: 5BBC09CB-DD5D-5B03-AFF9-1AF72318EF7B; **Taxon:** kingdom: Animalia; phylum: Annelida; class: Polychaeta; order: Phyllodocida; family: Syllidae; genus: Parasphaerosyllis; **Location:** continent: North America; waterBody: Gulf of California; country: Mexico; countryCode: MX; stateProvince: Baja California Sur; municipality: La Paz Bay; locality: San Lorenzo Channel; decimalLatitude: 24.386778; decimalLongitude: -110.315056; **Event:** eventDate: 05V2015; **Record Level:** institutionCode: UANL; collectionCode: NL-INV-0002-05-09

#### Travisia filamentosa

de León-González, 1998

0079D280-6642-59EF-B778-9FD7EE07060B

https://www.marinespecies.org/aphia.php?p=taxdetails&id=332635

##### Materials

- **Type status:** Holotype. **Occurrence:** catalogNumber: UANL 0264/1418; recordedBy: Jesús A. de León-González; individualCount: 188; occurrenceID: A778AFCB-1512-596D-9FF6-57833C46BB1F; **Taxon:** kingdom: Animalia; phylum: Annelida; class: Polychaeta; order: Scolecida; family: Travisiidae; genus: Travisia; **Location:** continent: North America; waterBody: Temperate Northern Pacific; country: Mexico; countryCode: MX; stateProvince: Baja California; locality: Western coast of Baja Peninsula; maximumDepthInMeters: 110; decimalLatitude: 25.911667; decimalLongitude: -112.865; **Event:** eventDate: 08X1987; **Record Level:** institutionCode: UANL; collectionCode: NL-INV-0002-05-09

## Analysis

The "Colección Poliquetológica de la Universidad Autónoma de Nuevo León" preserves type materials from 15 families (Capitellidae, Eunicidae, Flabelligeridae, Maldanidae, Micropthalmidae, Nereididae, Onuphidae, Orbiniidae, Oweniidae, Paraonidae, Pectinariidae, Pilargidae, Sabellidae, Syllidae and Travisiidae), 35 genera and 54 species of polychaetes. There are 41 holotypes, 187 paratypes (belonging to 43 species) and one syntype (Table 1). Most types are from Mexican localities (76%), the rest are from the Phillipines, New Caledonia, Japan, USA, Ecuador, El Salvador and Argentina.

## Supplementary Material

XML Treatment for Dasybranchethus pacifica

XML Treatment for Notodasus harrisae

XML Treatment for Notodasus hartmanae

XML Treatment for Notodasus kristiani

XML Treatment for Notodasus salazari

XML Treatment for Notomastus fauchaldi

XML Treatment for Notomastus landini

XML Treatment for Notomastus lobulatus

XML Treatment for Notomastus mazatlanensis

XML Treatment for Eunice chicasi

XML Treatment for Eunice romanvivesi

XML Treatment for Eunice salvadorensis

XML Treatment for Treadwellphysa veracruzensis

XML Treatment for Trophoniella reishi

XML Treatment for Trophoniella salazarae

XML Treatment for Clymenura scutata

XML Treatment for Struwela camposi

XML Treatment for Neanthes philippinensis

XML Treatment for Nereis baolingi

XML Treatment for Nereis casoae

XML Treatment for Nereis fauchaldi

XML Treatment for Nereis imajimai

XML Treatment for Nereis inflata

XML Treatment for Laeonereis watsoni

XML Treatment for Nicon orensanzi

XML Treatment for Nicon pettiboneae

XML Treatment for Perinereis rookeri

XML Treatment for Platynereis hutchingsae

XML Treatment for Platynereis mucronata

XML Treatment for Rullierinereis fauchaldi

XML Treatment for Stenoninereis tecolutlensis

XML Treatment for Websterinereis pettiboneae

XML Treatment for Australonuphis paxtonae

XML Treatment for Kinbergonuphis kristiani

XML Treatment for Paradiopatra barrazai

XML Treatment for Leitoscoloplos bajacaliforniensis

XML Treatment for Galathowenia kirkegaardi

XML Treatment for Aricidea (Aricidea) petacalcoensis

XML Treatment for Paradoneis magdalenaensis

XML Treatment for Paradoneis strelzovi

XML Treatment for Amphictene helenae

XML Treatment for Pectinaria santii

XML Treatment for Synelmis emiliae

XML Treatment for Acromegalomma schwindtae

XML Treatment for Chone orensanzi

XML Treatment for Claviramus kyushuensis

XML Treatment for Notaulax nigroincrustata

XML Treatment for Notaulax punctulata

XML Treatment for Notaulax salazari

XML Treatment for Parasabella yonowae

XML Treatment for Branchiosyllis riojai

XML Treatment for Branchiosyllis sanmartini

XML Treatment for Parasphaerosyllis irregulata

XML Treatment for Travisia filamentosa

A3616F2D-DB54-5F27-95B3-DC3A1FCDDB65 10.3897/BDJ.12.e118576.suppl1 Supplementary material 1 Protologues of type specimens UANL Data type References File: oo_996290.docx https://binary.pensoft.net/file/996290 García-Garza, de León-González and Tovar-Hernández

## Figures and Tables

**Table 1.** General data of type materials deposited at the "Colección Poliquetológica de la Universidad Autónoma de Nuevo León (México)".

**Taxon_Local_ID**	**Species**	**Holotype**	**Paratype**	**Syntype**	**Total**
	**Family Capitellidae Grube, 1862**				
**1**	*Dasybranchethuspacifica* García-Garza & de León-González, 2009	1	1		2
**2**	*Notodasusharrisae* García-Garza, Hernández-Valdez & de León-González, 2009	1			1
**3**	*Notodasushartmanae* García-Garza, Hernández-Valdez & de León-González, 2009	1	6		7
**4**	*Notodasuskristiani* García-Garza, Hernández-Valdez & de León-González, 2009	1	20		21
**5**	*Notodasussalazari* García-Garza, Hernández-Valdez & de León-González, 2009	1			1
**6**	*Notomastusfauchaldi* García-Garza & de León-González, 2015	1	2		3
**7**	*Notomastuslandini* García-Garza & de León-González, 2015	1	4		5
**8**	*Notomastuslobulatus* García-Garza & de León González, 2015	1			1
**9**	*Notomastusmazatlanensis* García-Garza, de León-González & Tovar-Hernández, 2019	1	4		5
	**Family Eunicidae Berthold, 1827**				
**10**	*Eunicechicasi* de León-González, Rivera & Romero, 2004	1			1
**11**	*Euniceromanvivesi* de León-González & Díaz Castañeda, 2006	1	2		3
**12**	*Eunicesalvadorensis* de León-González, Rivera & Romero, 2004	1			1
**13**	*Treadwellphysaveracruzensis* (de León-González & Díaz Castañeda, 2006)	1	7		8
	**Family Flabelligeridae de Saint-Joseph, 1894**				
**14**	*Trophoniellareishi* Salazar-Vallejo, 2012		1		1
**15**	*Trophoniellasalazarae* Salazar-Vallejo, 2012		7		7
	**Family Maldanidae Malogren, 1867**				
**16**	*Clymenurascutata* Tovar-Hernández & Yáñez-Rivera in Yáñez-Rivera et al. 2020		3		3
	**Family Microphthalmidae Hartmann-Schröder, 1971**				
**17**	*Struwelacamposi* Salazar-Vallejo, de León-González & Carrera-Parra, 2019	1	9		10
	**Family Nereididae Blainville, 1918**				
**18**	*Neanthesphilippinensis* de León-González & Salazar-Vallejo, 2003		3		3
**19**	*Nereisbaolingi* de León González & Solís-Weiss, 2000		1		1
**20**	*Nereiscasoae* de León-González & Solís-Weiss, 2001	1	6		7
**21**	*Nereisfauchaldi* de León-González & Díaz-Castañeda, 1998	1			1
**22**	*Nereisimajimai* de León González & Díaz-Castañeda, 1998	1			1
**23**	*Nereisinflata* de León González & Solís-Weiss, 2001		5		5
**24**	*Laeonereiswatsoni* de León-González, Méndez & Navedo, 2018	1	5		6
**25**	*Niconorensanzi* de León-González & Trovant, 2013		1		1
**26**	*Niconpettiboneae* de León-González & Salazar-Vallejo, 2003		1		1
**27**	*Perinereisrookeri* de León-González & Goethel, 2013	1	1		2
**28**	*Platynereishutchingsae* de León-González, Solís-Weiss & Valdez-Rocha, 2001	1	1		2
**29**	*Platynereismucronata* de León-González, Solís-Weiss & Valdez-Rocha, 2001	1			1
**30**	*Rullierinereisfauchaldi* de León-González & Solís-Weiss, 2000		1		1
**31**	*Stenoninereistecolutlensis* de León-González & Solís-Weiss, 1997		1		1
**32**	*Websterinereispettiboneae* de León-González & Balart, 2016	1			1
	**Family Onuphidae Kinberg, 1865**				
**33**	*Australonuphispaxtonae* de León-González, Cornejo-Rodríguez & Degraer, 2008	1	1		2
**34**	*Kinbergonuphiskristiani* de León-González, Rivera & Romero, 2004	1			1
**35**	*Paradiopatrabarrazai* de León-González, Rivera & Romero, 2004	1			1
	**Family Orbiniidae Hartman, 1942**				
**36**	*Leitoscoloplosbajacaliforniensis* de León-González & Rodríguez, 1996	1			1
	**Family Oweniidae Rioja, 1917**				
**37**	*Galathoweniakirkegaardi* de León-González & Sánchez-Hernández, 2012	1	10		11
	**Family Paraonidae Cerruti, 1909**				
**38**	Aricidea (Aricidea) petacalcoensis de León-González, Hernández-Guevara & Rodríguez-Valencia, 2006	1	3		4
**39**	*Cirrophorusmagdalenaensis* de León González, Hernández-Guevara & Rodríguez Valencia, 2006 = *Paradoneismagdalenaensis* (de León-González, Hernández-Guevara & Rodríguez-Valencia, 2006) *fide* Martínez (2019)	1	2		3
**40**	*Paradoneisstrelzovi* de León-González & Díaz-Castañeda, 2011	1	10		11
	**Family Pectinariidae de Quatrefages, 1866**				
**41**	*Amphictenehelenae* García-Garza & de León González, 2014	1			1
**42**	*Pectinariasantii* Tovar-Hernández & de León-González, 2022		7		7
	**Family Pilargidae Saint Joseph, 1899**				
**43**	*Synelmisemiliae* Salazar-Vallejo, 2003			1	1
	**Family Sabellidae Latreille, 1825**				
**44**	*Acromegalommaschwindtae* Tovar-Hernández, de León-González & Bybee, 2017	1	2		3
**45**	*Choneorensanzi* Tovar-Hernández, de León-González & Bybee, 2017	1	38		39
**46**	*Claviramuskyushuensis* Nishi, Tanaka & Tovar-Hernández, 2019		3		3
**47**	*Notaulaxnigroincrustata* Tovar-Hernández, García-Garza & de León-González, 2020	1	5		6
**48**	*Notaulaxpunctulata* Tovar-Hernández, García-Garza & de León-González, 2020	1	1		2
**49**	*Notaulaxsalazari* Tovar-Hernández, de León-González & Bybee, 2017	1	2		3
**50**	*Parasabellayonowae* Tovar-Hernández, de León-González & Bybee, 2017	1	3		4
	**Family Syllidae Grube, 1850**				
**51**	*Branchiosyllisriojai* Góngora-Garza, García-Garza & de León-González, 2011	1			1
**52**	*Branchiosyllissanmartini* Góngora-Garza, García-Garza & de León-González, 2011	1	5		6
**53**	*Parasphaerosyllisirregulata* Góngora-Garza, de León-González & Tovar-Hernández, 2024	1	3		4
	**Family Travisiidae Hartmann-Schröder, 1971**				
**54**	*Travisiafilamentosa* de León-González, 1998	1			1
		41	187	1	229
