# Supplementary material for: Catalogue of type specimens deposited in the Polychaeta Collection of the Universidad Autónoma de Nuevo Léon (Mexico)
Source: Biodivers Data J. 2024 Mar 12;12:e118576. doi: 10.3897/BDJ.12.e118576 (PMC10951615; doi:10.3897/BDJ.12.e118576)
Supplement: Supplementary material 1 — Protologues of type specimens UANL [file bdj-12-e118576-s001.docx]

Protologues of type specimens UANL

- de León-González JA, Rodríguez-Valencia JA (1996) Orbiniidae (Polychaeta) from soft bottom of the western coast of Baja California Peninsula, Mexico. Bulletin of Marine Science 59 (1): 169‑174. URL: <http://www.ingentaconnect.com/content/umrsmas/bullmar/1996/00000059/00000001/art00010?crawler=true>
- de León-González JA, Solís-Weiss V (1997) A new species of Stenoninereis (Polychaeta: Nereididae) from the Gulf of Mexico. Proceedings of the Biological Society of Washington 110 (2): 198‑202. URL: <https://www.biodiversitylibrary.org/page/35458224>
- de León-González JA (1998) Spionidae and Opheliidae (Annelida: Polychaeta) from the western coast of Baja California, Mexico. Bulletin of Marine Science 62 (1): 7‑16. [In English]. URL: <http://www.ingentaconnect.com/content/umrsmas/bullmar/1998/00000062/00000001/art00002>
- de León-González JA, Díaz-Castañeda V (1998) Two new species of Nereis (Polychaeta: Nereididae) from Todos Santos Bay, Ensenada, Baja California, Mexico. Proceedings of the Biological Society of Washington 111 (4): 823‑828. URL: <https://www.biodiversitylibrary.org/part/45525>
- de León-González JA, Solís-Weiss V (2000) A review of the polychaete family Nereididae from Western Mexico. Bulletin of Marine Science 67 (1): 549‑569. URL: <http://www.ingentaconnect.com/content/umrsmas/bullmar/2000/00000067/00000001/art00044>
- de León-González JA, Solís-Weiss V (2001) Two new species of Nereis (Polychaeta: Nereididae) from the Mexican Pacific. Proceedings of the Biological Society of Washington 114 (4): 881‑886. URL: <https://www.biodiversitylibrary.org/partpdf/43996>
- de León-González JA, Solís-Weiss V, Valadez-Rocha V (2001) Two new species of Platynereis (Polychaeta: Nereididae) from eastern Mexican shores. Proceedings of the Biological Society of Washington 114 (2): 389‑395. URL: <https://archive.org/details/biostor-84898>
- de León-González JA, Salazar-Vallejo SI (2003) Four new nereidid species (Annelida, Polychaeta) collected during the MUSORSTOM cruises in the Indo-Pacific Ocean. Zoosystema 25 (3): 365‑375. URL: <https://sciencepress.mnhn.fr/en/periodiques/zoosystema/25/3/quatre-nouvelles-especes-de-nereididae-annelida-polychaeta-des-campagnes-musorstom-dans-l-ocean-indo-pacifique>
- de León-González JA, Rivera CG, Romero MY (2004) Sublittoral Eunicidae and Onuphidae (Polychaeta) from soft bottom off El Salvador, eastern Pacific. Journal of the Marine Biological Association of the UK 84 (1): 93‑101. <https://doi.org/10.1017/s0025315404008951h>
- de León-González JA, Díaz-Castañeda V (2006) Eunicidae (Annelida: Polychaeta) associated with Phragmatopoma caudata Mörch, 1863 and some coral reefs from Veracruz, Gulf of Mexico. Scientia Marina 70 (3): 91‑99. URL: <https://doi.org/10.3989/scimar.2006.70s391>
- de León-González JA, Guevara NAH, Rodríguez-Valencia JA (2006) Paraonidae (Polychaeta) from western Mexico, with description of two new species. Journal of the Marine Biological Association of the United Kingdom 86 (2): 253‑262. <https://doi.org/10.1017/s0025315406013105>
- de León-González JA, Cornejo-Rodriguez MH, Degraer S (2008) A new species of Australonuphis (Polychaeta: Onuphidae) from the eastern Pacific. Journal of the Marine Biological Association of the United Kingdom 88 (4): 739‑742. <https://doi.org/10.1017/s0025315408001252>
- de León-González JA, Díaz-Castañeda V (2011) A new species of Paradoneis (Polychaeta: Paraonidae) from the western coast of Baja California, Mexico. Proceedings of the Biological Society of Washington 124 (1): 40‑44. <https://doi.org/10.2988/10-07.1>
- de León-González JA, Sanchez-Hernández V (2012) Galathowenia kirkegaardi sp. nov. (Polychaeta: Oweniidae) from the Gulf of Mexico. Journal of the Marine Biological Association of the United Kingdom 92 (5): 1013‑1017. <https://doi.org/10.1017/s0025315411000786>
- de León-González JA, Goethel C (2013) A new species of Perinereis (Polychaeta, Nereididae) from Florida, USA, with a key to all Perinereis from the American continent. ZooKeys 312: 1‑11. <https://doi.org/10.3897/zookeys.312.4535>
- de León-González JA, Trovant B (2013) A new species of Nicon Kinberg, 1866 (Polychaeta, Nereididae) from Ecuador, Eastern Pacific, with a key to all known species of the genus. ZooKeys 269: 67‑76. <https://doi.org/10.3897/zookeys.269.4003>
- de León-González JA, Balart E (2016) A new species of Websterinereis from the Gulf of California and redescription of Websterinereis foli (Fauvel, 1930) (Annelida, Nereididae). ZooKeys 614: 15‑26. <https://doi.org/10.3897/zookeys.614.8843>
- de León-González JA, Méndez N, Navedo JG (2017) Laeonereis watsoni (Annelida, Nereididae), a new species from western Mexico. Journal of the Marine Biological Association of the United Kingdom 98 (6): 1347‑1353. <https://doi.org/10.1017/s0025315417000583>
- García-Garza M, de León González JA (2014) A new species of Amphictene (Annelida, Pectinariidae) from the Gulf of Mexico, with a redescription of Amphictene guatemalensis (Nilsson, 1928). ZooKeys 367: 1‑9. <https://doi.org/10.3897/zookeys.367.6038>
- García-Garza M, de León-González J, Harris L (2017) Relocation of Dodecaseta McCammon & Stull, 1978 (Annelida, Capitellidae) in Notodasus Fauchald, 1972. ZooKeys 715: 93‑101. <https://doi.org/10.3897/zookeys.715.13936>
- García-Garza ME, de León-González JA (2009) A new species of Dasybranchethus (Annelida: Capitellidae) from Mexican Pacific, with a redescription of Dasybranchethus fauveli . Journal of the Marine Biological Association of the United Kingdom 89 (7): 1437‑1441. <https://doi.org/10.1017/s0025315409000241>
- García-Garza ME, Hernández-Valdez VD, De León-González JA (2009) Generic revision of Notodasus Fauchald, 1972 (Polychaeta: Capitellidae) with descriptions of four new species from the coasts of Mexico. Scientia Marina 73 (4): 809‑823. <https://doi.org/10.3989/scimar.2009.73n4809>
- García-Garza ME, de León-González JA (2015) The genus Notomastus (Polychaeta: Capitellidae) in the Gulf of California, Mexico, with the description of three new species. Proceedings of the Biological Society of Washington 128 (2): 176‑189. <https://doi.org/10.2988/0006-324x-128.2.176>
- García-Garza ME, de León-González JA, Tovar-Hernández MA (2019) Catalogue of Notomastus M. Sars, 1851 (Annelida, Capitellidae) and the description of a new species from the Gulf of California. Zootaxa 4577 (2). <https://doi.org/10.11646/zootaxa.4577.2.2>
- Góngora-Garza G, García-Garza ME, de León-González JA (2011) Two new species of Branchiosyllis (Polychaeta: Syllidae) from Western Mexico. Proceedings of the Biological Society of Washington 124 (4): 378‑385. <https://doi.org/10.2988/11-22.1>
- Góngora-Garza G, de León-González JA, Tovar-Hernández MA (2024) Redescription of Parasphaerosyllis indica Monro, 1937 (Annelida: Syllidae), with the establishment of a new species from Western Mexico. Biodiversity Data Journal 12: e116082. <https://doi.org/10.3897/BDJ.12.e116082>
- ICZN (1999) International Code of Zoological Nomenclature. 4. International Trust for Zoological Nomenclature, 306 pp.
- Martínez J (2019) Una nueva especie de Paradoneis Hartman, 1965 (Annelida: Paraonidae) procedente del NE de la península Ibérica (SE del golfo de Vizcaya, NE del océano Atlántico). Graellsia 75 (1). <https://doi.org/10.3989/graellsia.2019.v75.223>
- Molina-Acevedo I, Carrera-Parra L (2017) Revision of Marphysa de Quatrefages, 1865 and some species of Nicidion Kinberg, 1865 with the erection of a new genus (Polychaeta: Eunicidae) from the Grand Caribbean. Zootaxa 4241 (1). <https://doi.org/10.11646/zootaxa.4241.1.1>
- Nishi E, Tanaka K, Tovar-Hernández MA (2019) A new species of Claviramus (Annelida, Sabellida, Sabellidae) from the Ariake Inland Sea, Kyushu, Japan. ZooKeys 880: 25‑32. <https://doi.org/10.3897/zookeys.880.36281>
- Salazar-Vallejo S (2012) Revision of Trophoniella Hartman, 1959 (Polychaeta, Flabelligeridae). Zoosystema 34 (3): 453‑519. <https://doi.org/10.5252/z2012n3a1>
- Salazar-Vallejo S, de León-González JA, Carrera-Parra L (2019) Phylogeny of Microphthalminae Hartmann-Schröder, 1971, and revision of Hesionella Hartman, 1939, and Struwela Hartmann-Schröder, 1959 (Annelida, Errantia). PeerJ 7 <https://doi.org/10.7717/peerj.7723>
- Salazar-Vallejo SI (2003) Revision of Synelmis Chamberlin, 1919 (Annelida, Polychaeta, Pilargidae). Zoosystema 25 (1): 17‑42. URL: <http://sciencepress.mnhn.fr/en/periodiques/zoosystema/25/1/revision-de-synelmis-chamberlin-1919-annelida-polychaeta-pilargidae>
- Tovar-Hernández MA, de León-González JA, Bybee DR (2017) Sabellid worms from the Patagonian Shelf and Humboldt Current System (Annelida, Sabellidae): Phyllis Knight-Jones’ and José María Orensanz’s collections. Zootaxa 4283 (1). <https://doi.org/10.11646/zootaxa.4283.1.1>
- Tovar-Hernández MA, García-Garza ME, de León-González JA (2020) Sclerozoan and fouling sabellid worms (Annelida: Sabellidae) from Mexico with the establishment of two new species. Biodiversity Data Journal 8 <https://doi.org/10.3897/bdj.8.e57471>
- Tovar-Hernández MA, de León-González JA (2022) A new species of ice cream cone worm in the Gulf of California (Annelida, Pectinariidae). Biodiversity Data Journal 10 <https://doi.org/10.3897/bdj.10.e94772>
- Yáñez-Rivera B, Tovar-Hernández MA, Galván-Villa C, Ríos-Jara E (2020) Tubicolous polychaete worms (Annelida) from Bahía de Chamela Islands Sanctuary, Mexico, with the description of a new bamboo worm. Biodiversity Data Journal 8 <https://doi.org/10.3897/bdj.8.e57572>
